# A computational framework for cortical microtubule dynamics in realistically shaped plant cells

**DOI:** 10.1371/journal.pcbi.1005959

**Published:** 2018-02-02

**Authors:** Bandan Chakrabortty, Ikram Blilou, Ben Scheres, Bela M. Mulder

**Affiliations:** 1 Plant Developmental Biology, Wageningen University, Wageningen, The Netherlands; 2 Department of Living Matter, Institute AMOLF, Amsterdam, The Netherlands; 3 Laboratory of plant cell and developmental biology, King Abdullah University of Science and Technology, Thuwal, Kingdom of Saudi Arabia; 4 Cell Biology, Wageningen University, Wageningen, The Netherlands; University of California Irvine, UNITED STATES

## Abstract

Plant morphogenesis is strongly dependent on the directional growth and the subsequent oriented division of individual cells. It has been shown that the plant cortical microtubule array plays a key role in controlling both these processes. This ordered structure emerges as the collective result of stochastic interactions between large numbers of dynamic microtubules. To elucidate this complex self-organization process a number of analytical and computational approaches to study the dynamics of cortical microtubules have been proposed. To date, however, these models have been restricted to two dimensional planes or geometrically simple surfaces in three dimensions, which strongly limits their applicability as plant cells display a wide variety of shapes. This limitation is even more acute, as both local as well as global geometrical features of cells are expected to influence the overall organization of the array. Here we describe a framework for efficiently simulating microtubule dynamics on triangulated approximations of arbitrary three dimensional surfaces. This allows the study of microtubule array organization on realistic cell surfaces obtained by segmentation of microscopic images. We validate the framework against expected or known results for the spherical and cubical geometry. We then use it to systematically study the individual contributions of global geometry, cell-edge induced catastrophes and cell-face induced stability to array organization in a cuboidal geometry. Finally, we apply our framework to analyze the highly non-trivial geometry of leaf pavement cells of *Arabidopsis thaliana*, *Nicotiana benthamiana* and *Hedera helix*. We show that our simulations can predict multiple features of the microtubule array structure in these cells, revealing, among others, strong constraints on the orientation of division planes.

## Author summary

In contrast to animal cells, plant cells are encased in a rigid cell wall providing among others the necessary rigidity to contain the turgor pressure that allows the plant as a whole to raise itself against gravity. In order to maintain this mechanical integrity while growing requires plant cells to rigorously control their expansion. In this they are aided by a plant-unique intracellular structure called the cortical array, an organised set of long filamentous protein polymers called microtubules attached to the inside of the cell membrane. Experiments and modelling studies have revealed how collisions between these highly dynamic polymers promote the spontaneous alignment of microtubules observed in cells. However, it was hitherto unknown how the shape of cells influences this self-organisation process and helps in determining the overall orientation of the cortical array. The latter is key in deciding the axis of cell expansion and the orientation of cell division planes. Here we develop, validate and apply a simulation framework for studying the dynamics of interacting microtubules on arbitrary triangulated surfaces that can be extracted from three-dimensional reconstruction of real cells from confocal microscopy images. This allows us to start unravelling the delicate interplay between cell shape, effects of sharp cell-edges and developmental cues on the organisation of the cortical array.

## Introduction

It is well known that the cortical microtubule (hereafter abbreviated to MT) cytoskeleton in plant cells plays a decisive role in controlled cell expansion and oriented cell division, which together drive the plant morphogenesis [1]. In the absence of MT organizing centers like centrosomes [2], higher plants establish an ordered array of MTs at the cell cortex, the so-called cortical array (CA) [3]. Recent studies have revealed that cell shape may have influence on the orientation of the CA [4]. On the other hand, the orientation of the CA controls cell expansion and cell anisotropy, by guiding the deposition of cellulose synthase complexes along the MTs [5–8]. Through this coupling, the CA in turn can influence the cell shape, essentially setting up a morphogenetic feedback loop. This loop is possibly also amplified by a mechanical feedback mechanism discussed in [9]. Therefore, understanding both anisotropic cell expansion and oriented cell division, requires understanding the formation of the ordered CA from an initially disordered state just after cell division [10].

**Fig 1:**
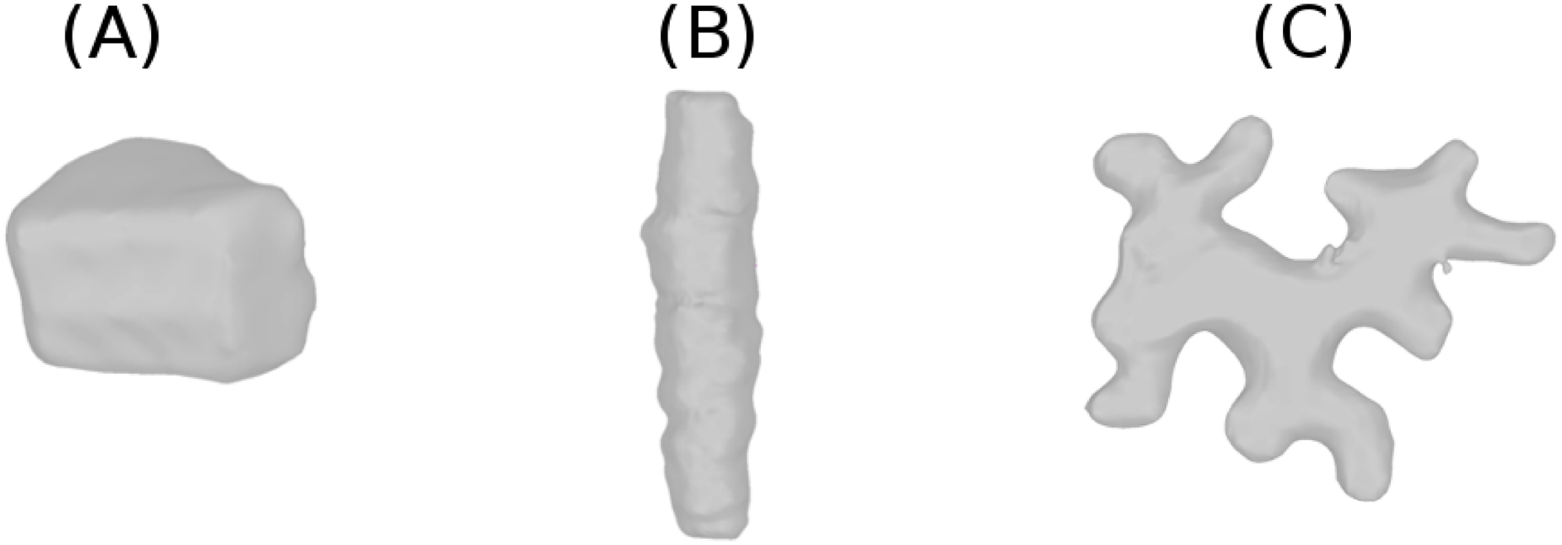
Plant cells with variety of shapes. (A) *Arabidopsis* root epidermal cell, (B) *Sorghum* leaf cell and (C) *Nicotiana* leaf cell.

For non-growing cells, the influence of cell shape on the formation of the CA will be static in nature and thus determined by geometrical features alone. A significant variety in these features is observed between different cell types (see Fig 1). For growing cells, the evolving cell shape may generate a corresponding dynamic influence on the CA formation process. If growth is slow compared to the collective dynamical time scale of the MTs, a quasi-static approach which samples cell shapes at different time points may still be a reasonable approximation.

**Fig 2:**
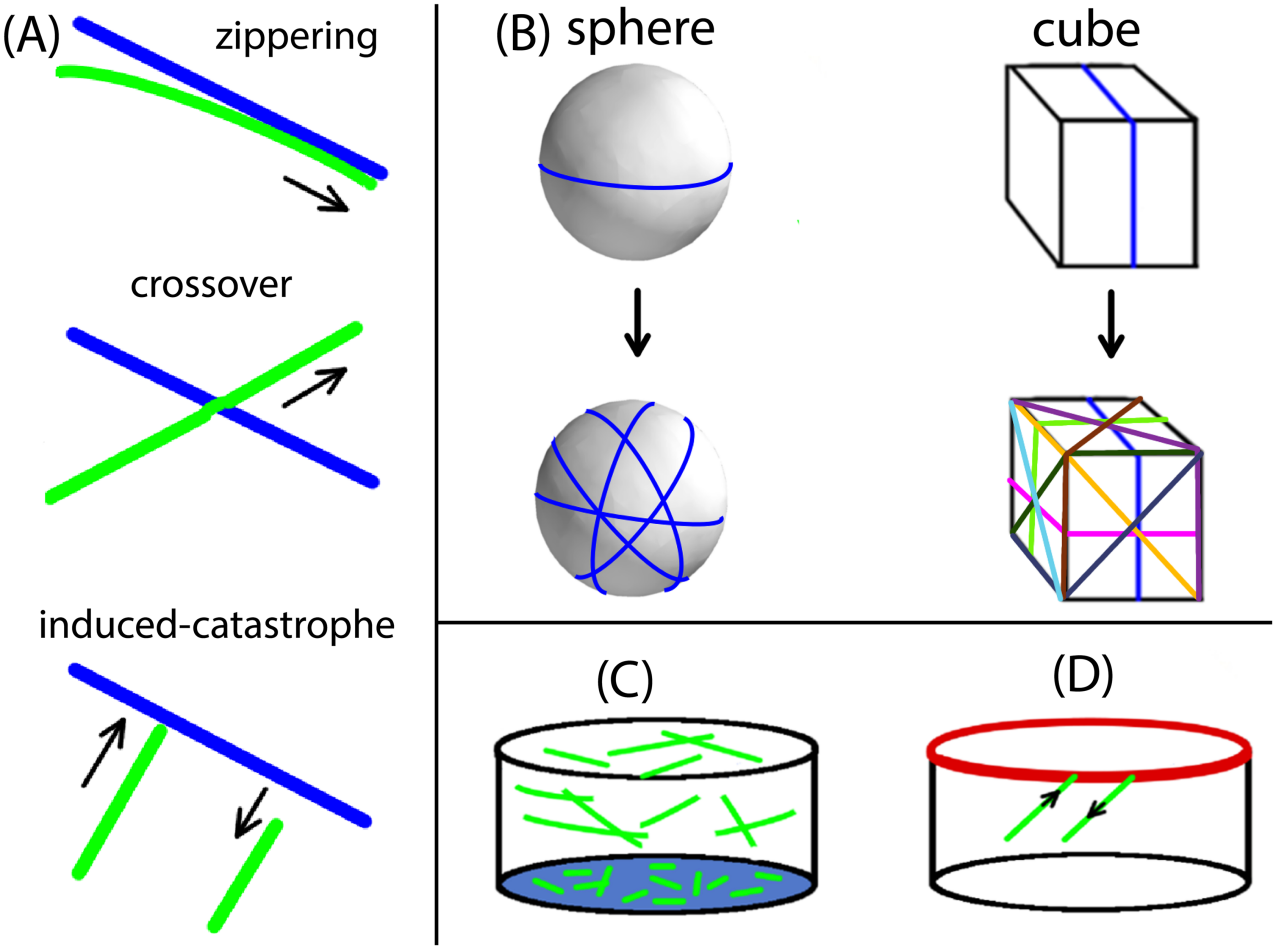
Different regulators of MT array formation. (A) Collision events during MT-MT interactions, where a steep angle of collision leads to *induced-catastrophe* or *crossover*, and shallow angle of collision leads to *zippering*.(B) Due to the attachment of the MTs to the two-dimensional cell surface (cortex), cell shape also influences MT interactions, hence MT order and array formation. On a spherical surface, an infinite number of unique directions to form closed geodesic paths are possible, suggesting an infinite number of possible paths of MT self-intersection. For a cubic surface, only nine unique directions to form closed geodesics are possible, suggesting a limited number of possible paths avoiding MT self-intersection. (C) Different degree of MT stabilization on different cell-faces may lead to variation in the average length distribution of MTs, i.e. domain with enhanced MT stabilization will have longer average MT length than the domain of less MT stabilization (coloured in faint blue). (D) At an edge between cell-faces (coloured in thick red), MTs undergo *edge-catastrophe* in a probabilistic manner, depending on the value of cell-edge angle.

MTs are highly dynamic and filamentous protein polymer aggregates, and form one of the principal components of the plant cytoskeleton [11]. MTs have structurally two distinct ends—a minus-end and a plus-end. The plus-end can dynamically switch from a growing state to a shrinking state or vice-versa. Switching of a MT plus-end from a growing state to a shrinking state is called catastrophe while the reverse switching of a shrinking state to a growing state is called rescue. This phenomenon of reversible switching of MT plus-ends between two states is called dynamic instability. On average, the minus-end of an unstabilized MT continually is in a shrinking state. Thus, the combination of overall growth at the plus-end and shrinkage at the minus-end seemingly moves a MT as a whole. This motion is called treadmilling and has been observed in both in vitro [12–14] and in vivo [15]. In contrast to animal cells, plant cells do not have a well defined MT organizing center. Instead MT activity is dispersed over the whole cell cortex, driven by the localized nucleation of new MTs by *γ*-tubulin complexes [16]. The cortical MTs are confined to a thin layer of cytoplasm just inside the plasma membrane of the plant cell and are attached to the cell envelope, ensuring that MTs do not translate or rotate as a whole [17, 18]. In spite of their fixed attachment to the cell cortex, cortical MTs do show mobility which is due to treadmilling motion [17, 19]. Two dimensional attachment to the cell cortex allows MTs to interact with each other via collisions, which occur when the polymerizing plus-end of a growing MT encounters a pre-existing MT. Depending on the value of the collision angle, three different possible events are observed [20]. For shallow angles (≲ 40^0^), a growing MT bends toward the direction of the MT encountered and this kind of adaptive event is called *zippering*. For steeper angles (≳ 40^0^), the encounter may lead to a so-called *induced-catastrophe*, where the initially growing MT switches to a shrinking state. Alternatively, the growing MT may slip over the one encountered, leading to a *crossover* event (see Fig 2). In vivo imaging of cortical MTs has revealed that they nucleate at the cortex, either from isolated nucleation complexes or from pre-existing MTs, and gradually develop from an initially sparse and disorganized state into a final ordered array over a time period of an hour [17, 18, 21–24] after the previous cytokinesis. MTs have a finite lifetime and ultimately *disappear* by shrinking to zero length. Plant cells within tissues typically have well defined relatively flat faces, bordered by cell-edges of significantly higher curvature [25]. In root epithelial cells microtubules have been observed to undergo catastrophes when they encounter these cell-edges with a probability that increases with increasing curvature of the cell-edge [26]. We will denote these as *edge-catastrophe* events.

While existing experimental studies inform us about the molecular events and key parameters that are involved in CA formation [17, 19, 24, 27, 28], it represents a complex emergent phenomenon that is the macroscopic result of a large number of stochastic microscopic events involving large numbers of MTs. For a full mechanistic understanding of biological processes of this type, mathematical and computational modelling has by now been recognised as indispensable [29]. From the outset, starting with the seminal work of Dixit & Cyr [20], attempts have been made to model CA formation (for reviews on the different approaches involved please consult [30, 31]). From this work a consensus hypothesis has emerged to explain the spontaneous order observed in the CA. This mechanism, also dubbed ‘survival of the aligned’ [32], can account for the statistically robust spontaneous alignement of MTs under well-characterized conditions, and appears to be consistent with observations. We therefore adopt this hypothesis as the starting point in the present work.

As far as the influence of cell geometry on CA formation is concerned, modelling studies have so far been limited to two dimensional (2D) approaches where the effect of a closed shape in three dimension (3D) was mimicked by boundary conditions [33], or geometrically simple shapes where boundaries are readily described by mathematical expressions, such as cubes [34] and cylinders [35]. Also, some of the simulations reported relied on finite time step brownian dynamics, at most using a Gillespie-type event-sampling algorithm [36] for the spontaneous changes in MT dynamical state. This severely limits the time-efficiency of these simulations, and makes the acquisition of results for wide ranges of parameters with sufficient statistics a very difficult task. Tindemans et al. [35] therefore developed a fully event-driven algorithm (for a general introduction see [37], Chapter 6.1), in which not only the spontaneous stochastic state changes but also the collisions between MTs are performed by directly sampling from a dynamic list of potential future collision events and their associated waiting times, which is constantly updated. This achieves a speed up of at least three orders of magnitude with respect to standard finite time step algorithms, and eliminates errors in the location of the collision points as well.

Here we show how the algorithm of [35] can be implemented efficiently on arbitrary triangulated surfaces, such as those obtained by segmenting 3D reconstructed confocal microscopy images of cells. Strikingly, the additional computational overhead incurred by having to determine the correct passage from triangle to triangle in a manner consistent with the 3D path, is almost completely balanced by the speed up of the collision predicting algorithm due to the strong localization of the search area. In this way the new implementation is almost as fast as the original implementation, and allows the rapid exploration of many different parameter settings and/or geometries each sampled independently many times to obtain statistically reliable averaging over the stochastic ensemble. In addition, the MT dynamical parameters can be chosen independently within each triangular domain and passage probabilities can be associated with the shared edges between the triangular domains. This allows us to easily implement biologically relevant effects such as the observed *edge-catastrophes* at locations of strong curvature, or potential differences in local MT dynamics due to developmental distinctions between cell-faces, e.g. between faces created by recent cell divisions and faces that have expanded by growth.

## Methods

Here we outline our computational method for simulating MT dynamics on surfaces of arbitrary shape (see Fig 1). First, we describe the processing of confocal laser scanning microscopy images of plant cells, transforming them into a triangulated surface mesh that can be directly used as simulation input. Next, we describe the implementation of the simulations and the order parameter that we define to measure the degree of MT order and the associated array orientation. Finally, we discuss how the working set of parameters for our simulations was selected.

### Processing confocal images for simulation

**Fig 3:**
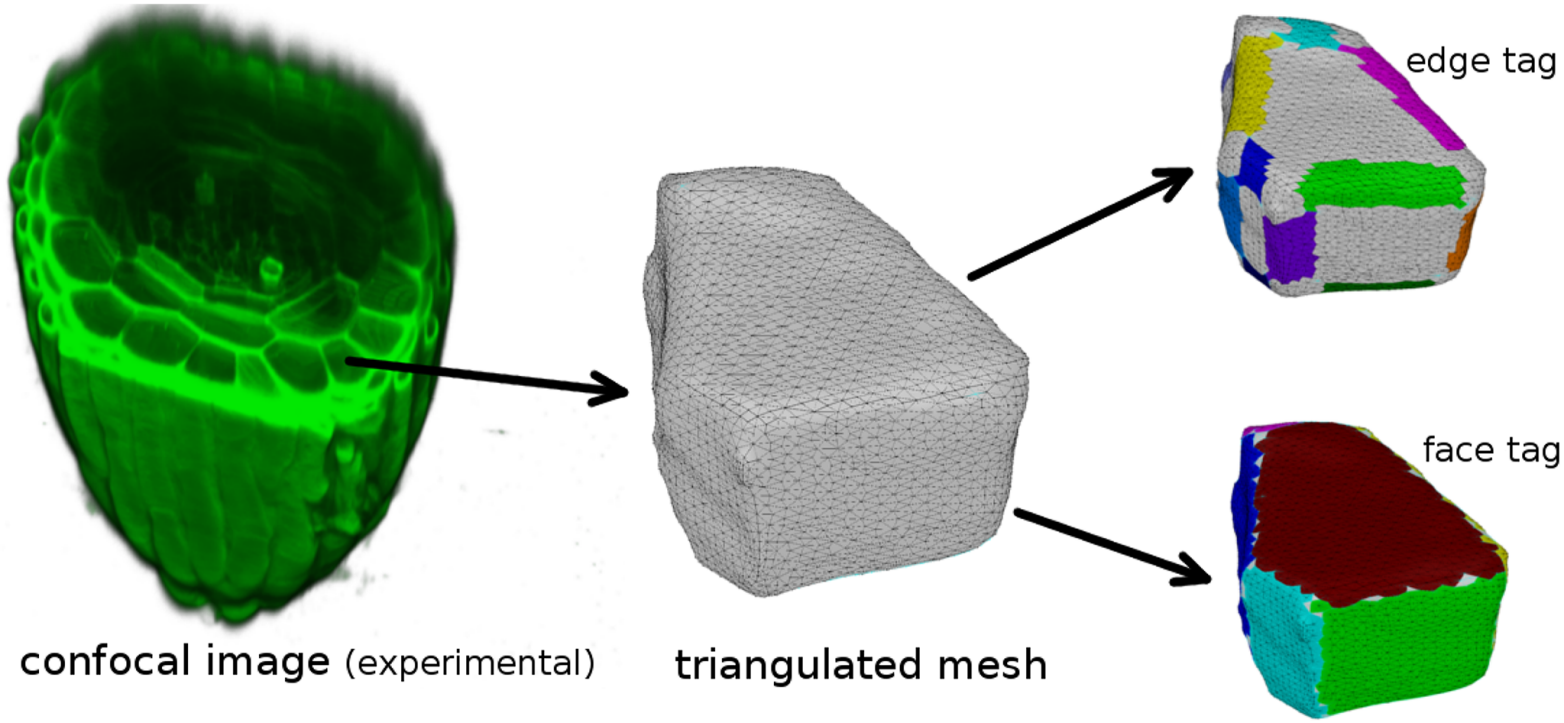
Image processing steps in transforming confocal image of a cell of *Arabidopsis thaliana* root apex into a triangulated surface mesh, which is the basic requirement as input for MT simulation. Confocal image courtesy: Viola Willemsen (WUR).

In Fig 3, we illustrate the procedure of transforming experimentally obtained confocal image into simulation input. First, using the image processing software *morphographX* [38], we segment the experimentally obtained confocal images and extract the different cell shapes. For segmentation, confocal image stacks (tif) were Gaussian blurred using sigma value 0.3 *μm*, subsequently we applied the ITK watershed auto-seeding with level threshold value in the range 500–1500. Then, we approximated the segmented cells by creating triangulated surface meshes using marching cubes 3D with cube size in the range 0.5–1.0 *μm*. Using the surface mesh processing software *meshLab* [39], we tag the different cell-edges and cell-faces, which are represented by appropriately coloured associated triangles. Finally, all this information is stored in a single mesh file, where each triangle has a cell-edge tag with the values of the associated cell-edge angles and cell-face tags (see S1 File, Sec. SI.1).

### Simulation algorithm

Confinement of the dynamics of cortical MTs to the surface (cortex) of plant cell effectively reduces the system to two spatial dimensions. In our modelling framework, we exploit this advantage by treating the cell shape as a 2D surface, embedded in the ambient 3D space. Because we are working with a triangulated approximation of surfaces, we developed an algorithm to establish a connectivity graph of the triangles [40] in order to appropriately propagate MT dynamics between adjacent triangles.

**Fig 4:**
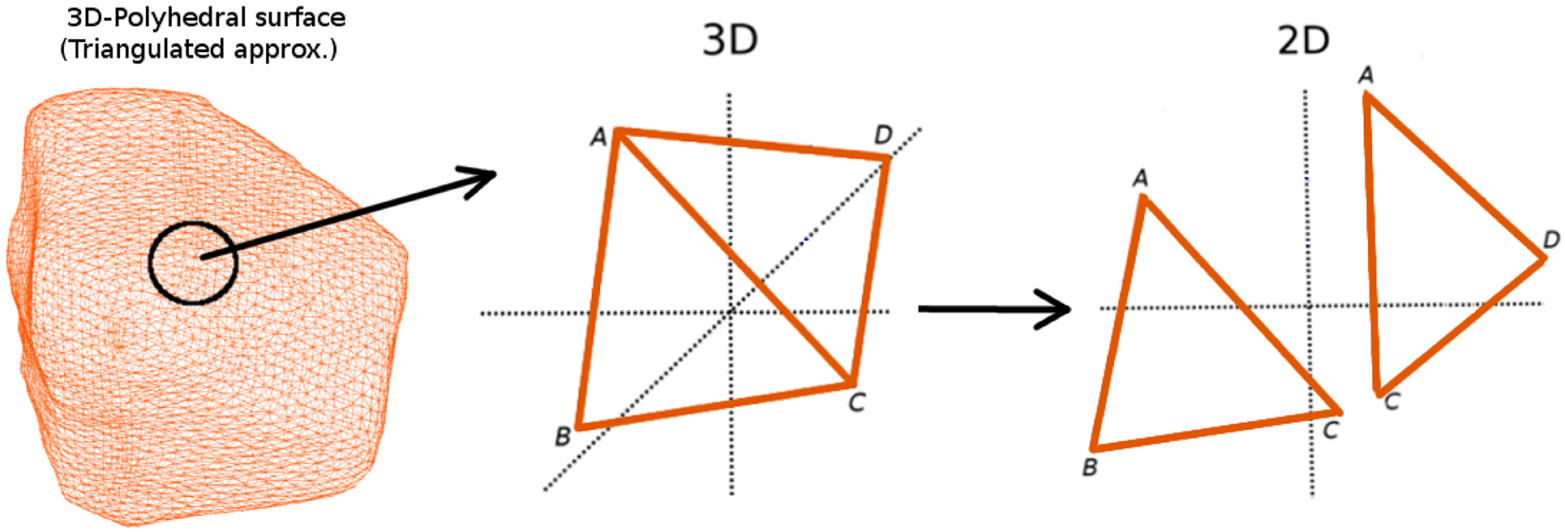
Mapping of triangles from the 3D surface mesh to the 2D plane. Δ*ABC* and Δ*ADC* are connected through a shared edge *AC* in the 3D *x* − *y* − *z* space, but upon quaternion rotation operation end up separated in the 2D *x* − *y* plane.

Using a combination of rotations, implemented by quaternions [41], and *z*-axis translation, we first transform the 3D-triangulated surface from the 3D *x* − *y* − *z* space to the 2D *x* − *y* plane (see Fig 4). Each individual triangle has a unique quaternion operator which is fully determined by its normal vector in 3D, which operates on all its vertices simultaneously. Different triangles have different normals, and hence end up in different, typically disjoint, 2D positions after rotation, irrespective of whether they had shared edges or not, but this position is irrelevant to the dynamics within each triangle.

**Fig 5:**
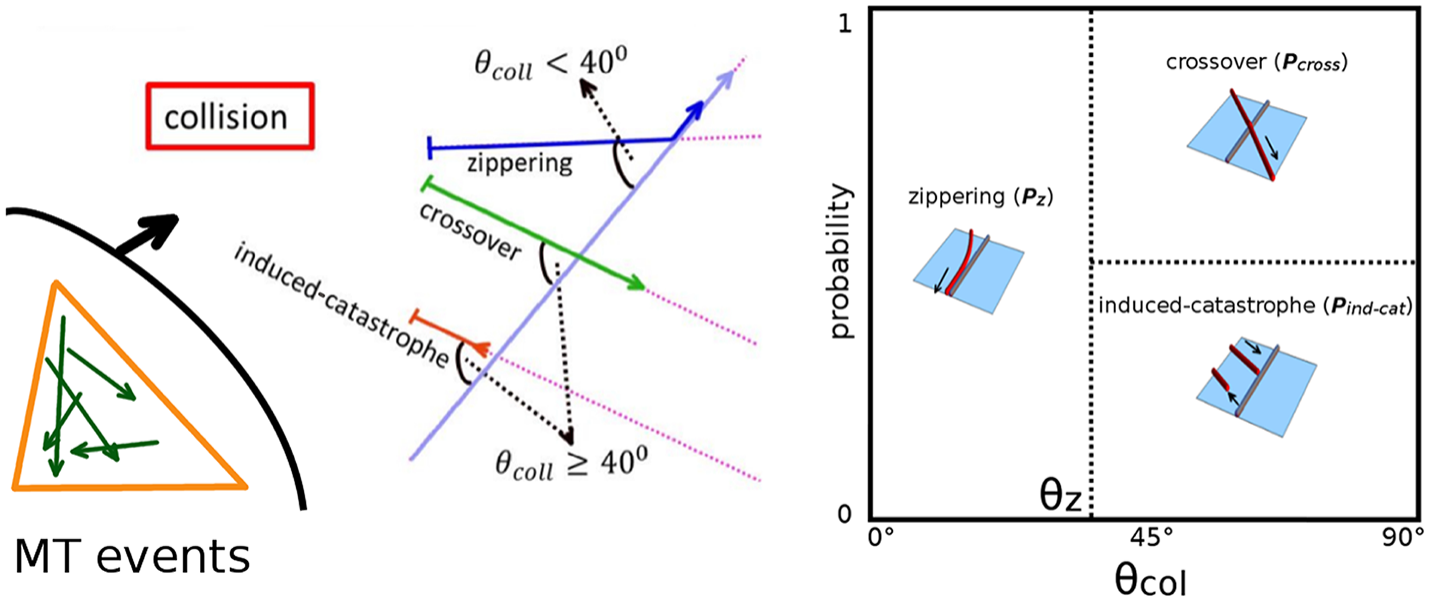
Simulation of MT events on an individual triangle. Upon nucleation, a straight line trajectory is assigned as a track for growth. Dynamic instabilities of MT tips are included through specification of *spontaneous-catastrophe* and rescue rates. Type of collision events, i.e. *zippering*, *induced-catastrophe* and *crossover*, is determined on basis of the intersection angle between MT trajectories.

We simulate MT nucleation, growth, shrinkage and collision events within each individual triangle (see Fig 5). In order to nucleate new MTs on the surface, we first randomly select a triangle from the entire set and generate uniformly distributed points within the selected triangle [42]. The initial MT growth direction is made isotropic by choosing a random angle of nucleation in the range [0, 2*π*].

The persistence length of a MT is much longer than its average length, typically of the order of a few *μ*m’s, [43, 44] and therefore much larger than the typical dimensions of our triangles (well below 1*μm*), allowing us to model the individual MT within a triangle as an elongating straight line segment. The location of the collision point between two MTs is then readily determined by calculating the intersection point of their respective trajectories [45, 46]. To deal with the various MT events, we implement an event-driven simulation technique [35]. The events are separated into two categories: stochastic events and deterministic events. The stochastic events associated with MTs are independent and result from Poissonian random processes specified by the nucleation rate *r*_*n*_, the *spontaneous-catastrophe* rate *r*_*c*_ and the rescue rate *r*_*r*_. The deterministic events associated with the MTs include collisions, disappearance, intersection with triangle-edges, and simulation control events (i.e extracting simulation output at fixed time intervals). In between events, the plus-ends of MTs either grow or shrink with velocities *v*_+_ and *v*_−_ respectively, while the minus-end retracts with the treadmilling speed *v*_*tm*_, so that all length changes between events are readily computed.

**Fig 6:**
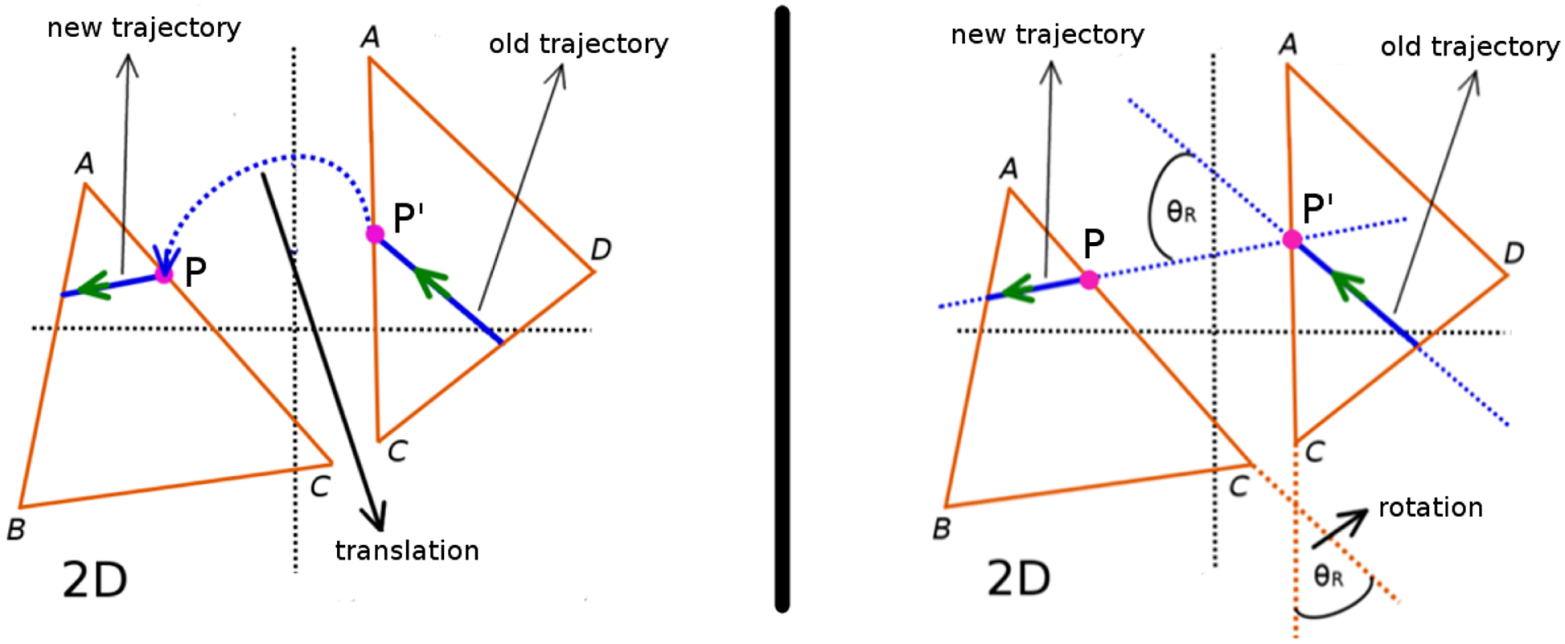
Propagating MT dynamics between neigboring triangles. An affine transformation is used to translate a growing MT tip from edge *AC*_(Δ*ADC*)_ of Δ*ADC* to edge *AC*_(Δ*ABC*)_ of Δ*ABC* (left panel). Upon translation, the MT growth direction is rotated by an angle *θ*_*R*_ (see Eq 1) that equals the angle between *AC*_(Δ*ADC*)_ and *AC*_(Δ*ABC*)_. A new trajectory along this rotated direction is then generated for the MT to continue its dynamics on Δ*ABC* (right panel).

We use triangle-edge to triangle-edge links (obtained from the connectivity graph) to propagate MT dynamics from one triangle to its relevant neighbour. For example, in Fig 6 Δ*ABC* is connected with Δ*ADC* through the shared edge *AC*, which are linked. The proper transition of a MT from Δ*ADC* to Δ*ABC* through the edge *AC* on the 3D surface requires, in the 2D plane,

1. *Translation* of the associated growing MT tip from edge *AC*_(Δ*ADC*)_ of Δ*ADC* to edge *AC*_(Δ*ABC*)_ of Δ*ABC*.
2. *Rotation* of the MT trajectory from Δ*ADC* to Δ*ABC*.

Using an affine transformation [47] on the coordinates of the growing MT plus-end tip at point *P*′(*x*′, *y*′) of edge *AC*_(Δ*ADC*)_, we *translate* (see Fig 6 left panel) the tip to the edge *AC*_(Δ*ABC*)_. Next, we *rotate* (see Fig 6 right panel) the MT trajectory with rotation angle,

$$\begin{array}{c} \theta_{R}=\arccos(\frac{{\vec{AC}}_{\left(\Delta ABC\right)}.{\vec{AC}}_{\left(\Delta ADC\right)}}{|\vec{AC}{|}^{2}}) \end{array}$$ (1)

This rotation in the 2D plane implements the assumption that a MT grows along the straightest possible path on the 3D surface, also known as a geodesic. Although the mechanical energy of bending could conceivably also play a role in determining the path of the MT, potentially causing a more complex coupling to the geometry, we have, in the absence of sufficient experimental data discriminating these effects, chosen to opt for this arguably simplest rule.

During transition of a MT from one triangle to another triangle, we implement the probability of local *edge-catastrophe* based on the local value of triangle-edge angle between the adjacent triangles (see S1 File, Sec. SI.2). We also use a cell-face tag, which is assigned to the triangles during the input surface mesh creation (see S1 File, Sec. SI.3), to implement local differences in stability of MTs e.g. by changing the local *spontaneous-catastrophe* rate. More generically, our simulation framework allows arbitrary domain specific parametrisation, with the smallest domain being a single triangle. This flexibility allows full freedom to incorporate experimental observations on local MT dynamics or test additional relevant hypotheses.

**Fig 7:**
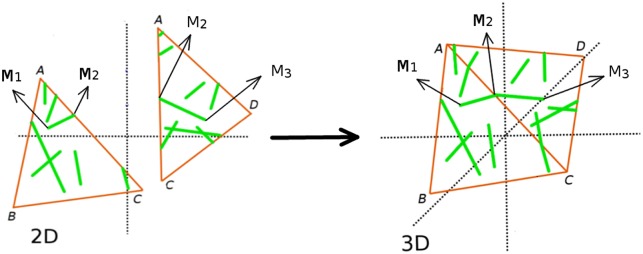
Mapping MT segments from the 2D plane back to the 3D surface. The inverse quaternion rotation operator of Δ*ABC* is applied to the MT segment $\bar{M_{1}M_{2}}$, and a similar operation is performed on MT segment $\bar{M_{2}M_{3}}$ of Δ*ADC*. If Δ*ABC* and Δ*ADC* are connected to each other via the edge *AC*, the MT segments $\bar{M_{1}M_{2}}$ and $\bar{M_{2}M_{3}}$ also get reconnected in their 3D position.

Finally, whenever required we apply the inverse quaternion transformation on the set of 2D triangles and the MT segments present on them, to reconstruct the 3D surface with the MT configurations realised. Through this transformation, segments of a MT which appear to be disconnected in the set of 2D triangles, get (re)connected on the 3D surface. As illustrated in Fig 7, the segment $\bar{M_{1}M_{2}}$ is attached to Δ*ABC* and the segment $\bar{M_{2}M_{3}}$ is attached to Δ*ADC* in the 2D plane. After the inverse quaternion mapping, these two segments get connected in the 3D surface and represent a single MT with end points (*M*_1_, *M*_3_).

**Fig 8:**
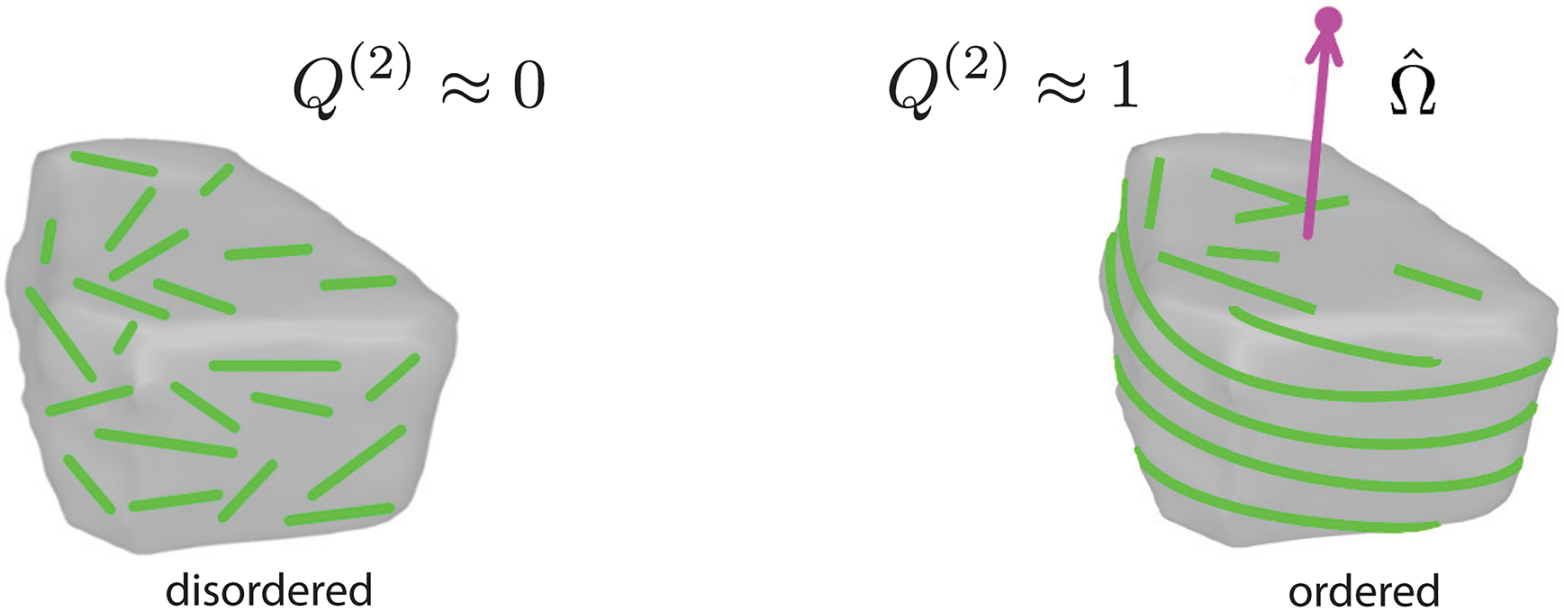
Schematic representation of the global order parameter. The scalar part *Q*^(2)^ measures the degree of MT order. The vector part $\hat{\Omega }$ measures the orientation of the associated MT array and is perpendicular to the plane of the MT array (magenta arrow). The dot (a filled magenta circle) at the tip of $\hat{\Omega }$ provides a concise representation of the MT array orientation.

To measure the degree of order and the orientation of the MT array, we define two order parameters. The first parameter is a scalar (*Q*^(2)^), which measures the average *degree* of ordering of the MTs, and the second a vector $\hat{\Omega }$, which indicates the global *orientation* of the array. Both these parameters are derived from a single tensorial order parameter **Q**^(2)^ (see S1 File, Sec. SI.4), *Q*^(2)^ being the absolute value of the smallest eigenvalue of this tensor and $\hat{\Omega }$ the corresponding normalized eigenvector, i.e. $|\hat{\Omega }|=1$. For completely random orientation of MTs *Q*^(2)^ ≈ 0 and for well organised MTs that form an array, *Q*^(2)^ ⪅ 1. The direction of $\hat{\Omega }$ is *perpendicular* to the average local orientation of the MTs (see Fig 8), i.e. perpendicular to the plane onto which the total projection of individual MTs is maximal on average.

**Fig 9:**
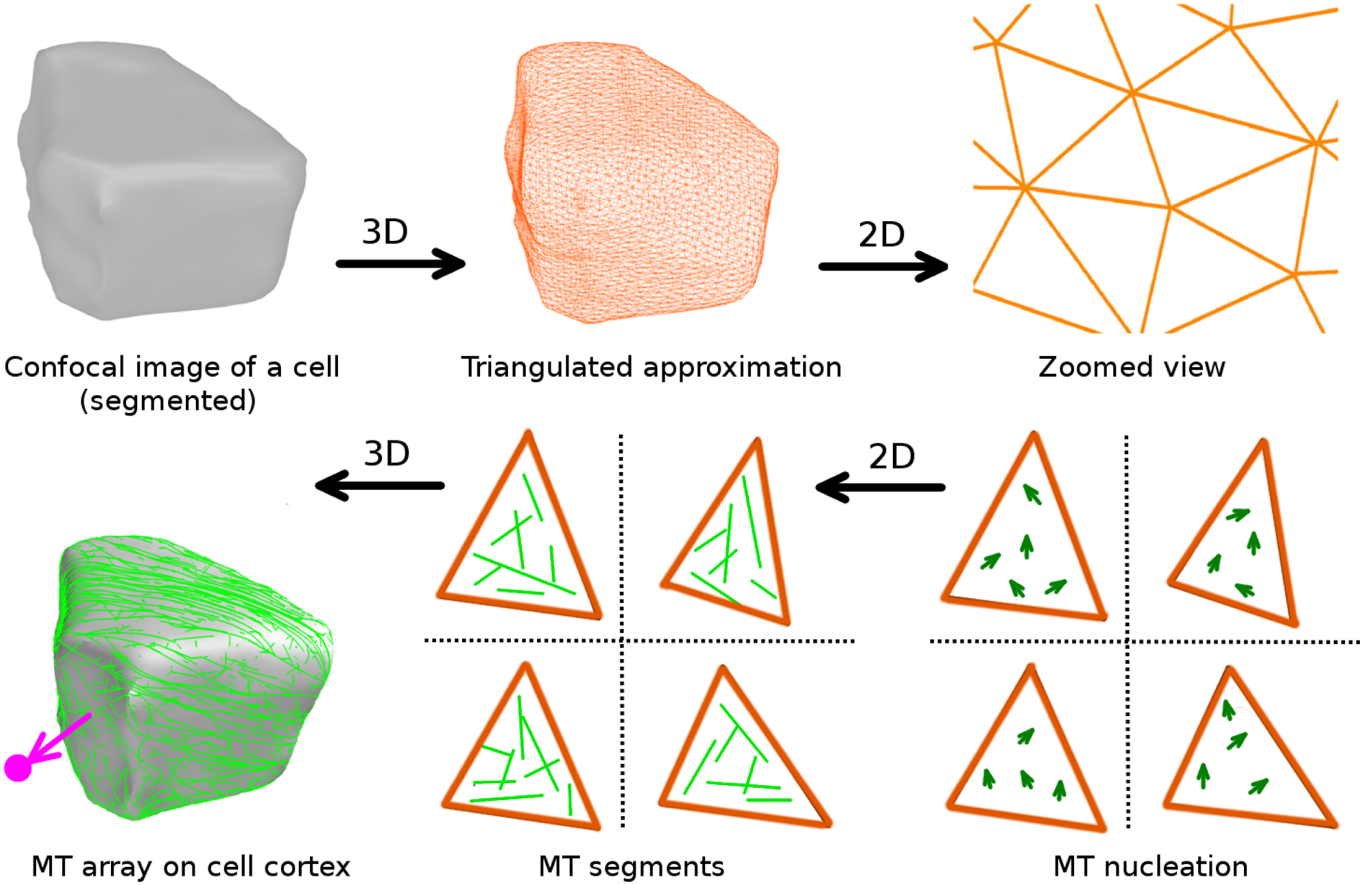
A schematic overview of the workflow in our simulation approach.

In Fig 9, we present an overview of the entire simulation approach.

### Working domain of parameter values for simulation

In order to obtain a suitable set of MT dynamical parameters for our simulations, we make use of the control parameter *G*, introduced and used in [48, 49]. This parameter encapsulates the effect of all six single MT dynamics parameters (speeds *v*_+_, *v*_−_ and *v*_*tm*_, dynamical instability rates *r*_*c*_, *r*_*r*_ and nucleation rate *r*_*n*_) into a single magnitude that controls the frequency of MT interactions which are required to ensure a spontaneously ordered state. Its explicit form identifies it as the ratio between two length scales

$$\begin{array}{c} G=-\frac{{\left(\frac{2\left(v_{+}-v_{tm}\right)\left(v_{-}+v_{tm}\right)}{r_{n}\left(v_{+}+v_{-}\right)}\right)}^{\frac{1}{3}}}{{\left(\frac{r_{r}}{v_{-}+v_{tm}}-\frac{r_{c}}{v_{+}-v_{tm}}\right)}^{-1}}=-\frac{l_{0}}{l_{avg}}. \end{array}$$ (2)

**Fig 10:**
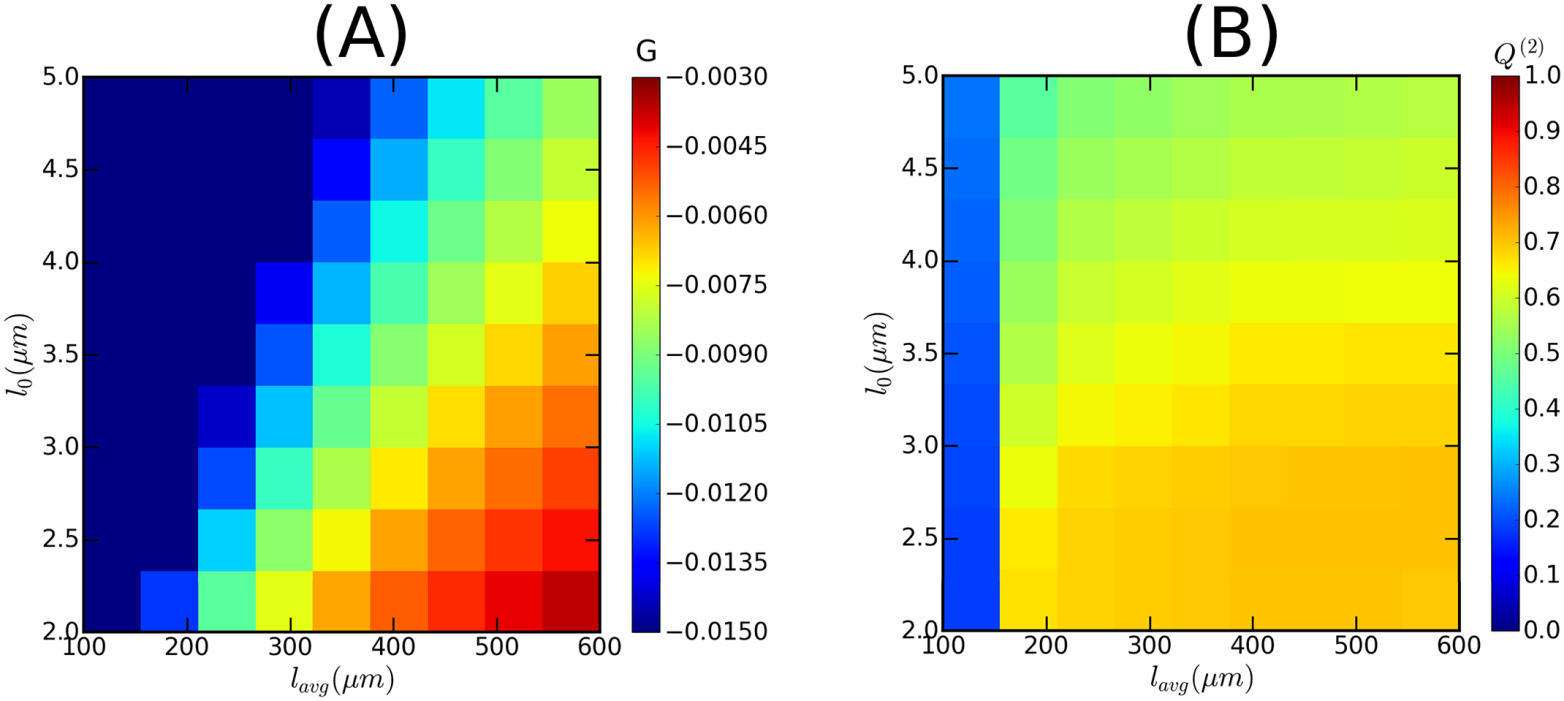
Working domain of MT simulation. Heat map showing: (A) values of MT-MT interaction control parameter *G*, and (B) values of the global scalar order parameter *Q*^(2)^. A value of *G* ⪆ −0.005 assured sufficient MT-MT interaction, leading the system to achieve *Q*^(2)^ ≳ 0.70 (for the time evolution, refer to S1 File, Fig S1.5). The values of *G* were calculated by using the MT dynamics parameter values described in [49], except the nucleation rate *r*_*n*_ (see S1 File, Sec SI.5). To assure sufficient number of MTs, we choose *r*_*n*_ ≈ 0.01 *sec*−1 *μm*^−2^. These simulations were performed using a tubulin density *ρ*_*tub*_ = 10 *μm*^−1^, defined as the ratio of the total length of MT that could be created from the available monomers to the total surface area of the shape.

Here *l*_0_ is the MT-MT interaction length, and *l*_*avg*_ is the average length of MTs in the absence of any interaction effects. The statement that *G* is the single control parameter was only strictly derived for spatially homogeneous and open, boundary-free domains. To allow for possible effects of a closed cell geometry, we chose to independently vary the two factors involved: *l*_0_ by varying the MT nucleation rate *r*_*n*_ and *l*_*avg*_ through varying the *spontaneous-catastrophe* rate *r*_*c*_. Moreover, and unless explicitly mentioned, we implement homeostatic control of the MT growth speed *v*_+_ by implementing a finite tubulin pool (see S1 File, Sec. SI.6 for details), as this speeds up the relaxation towards a steady state. The remaining simulation parameters are taken from [35]. We tested the parameter choice on a spherical surface with radius *r* ≈ 6 *μm*, inspired by the typical dimensions of a *Arabidopsis thaliana* embryonic cell. This is at the smaller end of the spectrum of plant cell sizes and therefore the most stringent test on finite size effects. We found that we could obtain robustly ordered arrays with *Q*^(2)^ ≳ 0.70 at a state point with *l*_0_ = 2.05 *μm* and *l*_*avg*_ = 417.5 *μm*, corresponding to *G* ≈ −0.005 (see Fig 10). The fact that *G* < 0 ensures bounded growth regime, where the length of MTs is always finite. All parameters used are summarised in S1 File, Sec. SI.5 (Table 1).

It is important to note that *l*_*avg*_ is the theoretically expected value of average MT length assuming absence of any interaction effects. However, in simulation MTs will interact with each other leading to changes in effective values of MT dynamics parameters, i.e. modification in the values of over all catastrophe rates due to effect of *induced-catastrophe*. These dynamic changes in MT dynamics parameter will also modify the average MT length to a value $\bar{l}$ different from *l*_*avg*_. In the coming sections, average MT length will correspond to the simulated value of average MT length, i.e. $\bar{l}$. For further clarification on this point, variation in the value of $\bar{l}$ with respect to *l*_*avg*_ is described in S1 File, Sec. SI.6.

Finally, we did not take any cell cycle related variations in MT dynamics into account, as this work focusses on understanding the relative contributions of the basic MT-MT interaction rule, shape anisotropy, *edge-catastrophe* and differential MT stabilization at developmentally different cell-faces on CA formation in interphase. We note, however, that such time-dependent effects could readily be implemented within our framework.

### Implementation of simulations

The simulation code is written in C++, and compiled using GNU GCC 4.8.4 compiler. Development and incidental runs were done on a standard PC equipped with Linux. Production runs were performed on the Dutch National Grid infrastructure comprising 10000 distributed compute nodes. Individual runs of 10 hours of biological time took between 20 seconds to 120 seconds wall clock time, depending on the parameter settings.

## Results

### Validation of the framework

We performed a set of simulations validating our computational framework. We first tested to what extent the triangulation of otherwise smooth surfaces influences the final results. To do so we considered the case of approximating a perfect sphere, varying both the number of triangles employed, as well as the triangulation method. The key desiderata in this case are (i) that in steady state, due to the spherical symmetry, there should be no bias in the orientation $\hat{\Omega }$ of the ordered array, and (ii) that the value of the scalar order parameter *Q*^(2)^ does not depend on the nature of the triangulation. Next, in order to test the system as a whole against a known, and geometrically non-trivial case, we reproduced previously reported results on a cubic surface [50].

#### Effect of triangulation

**Fig 11:**
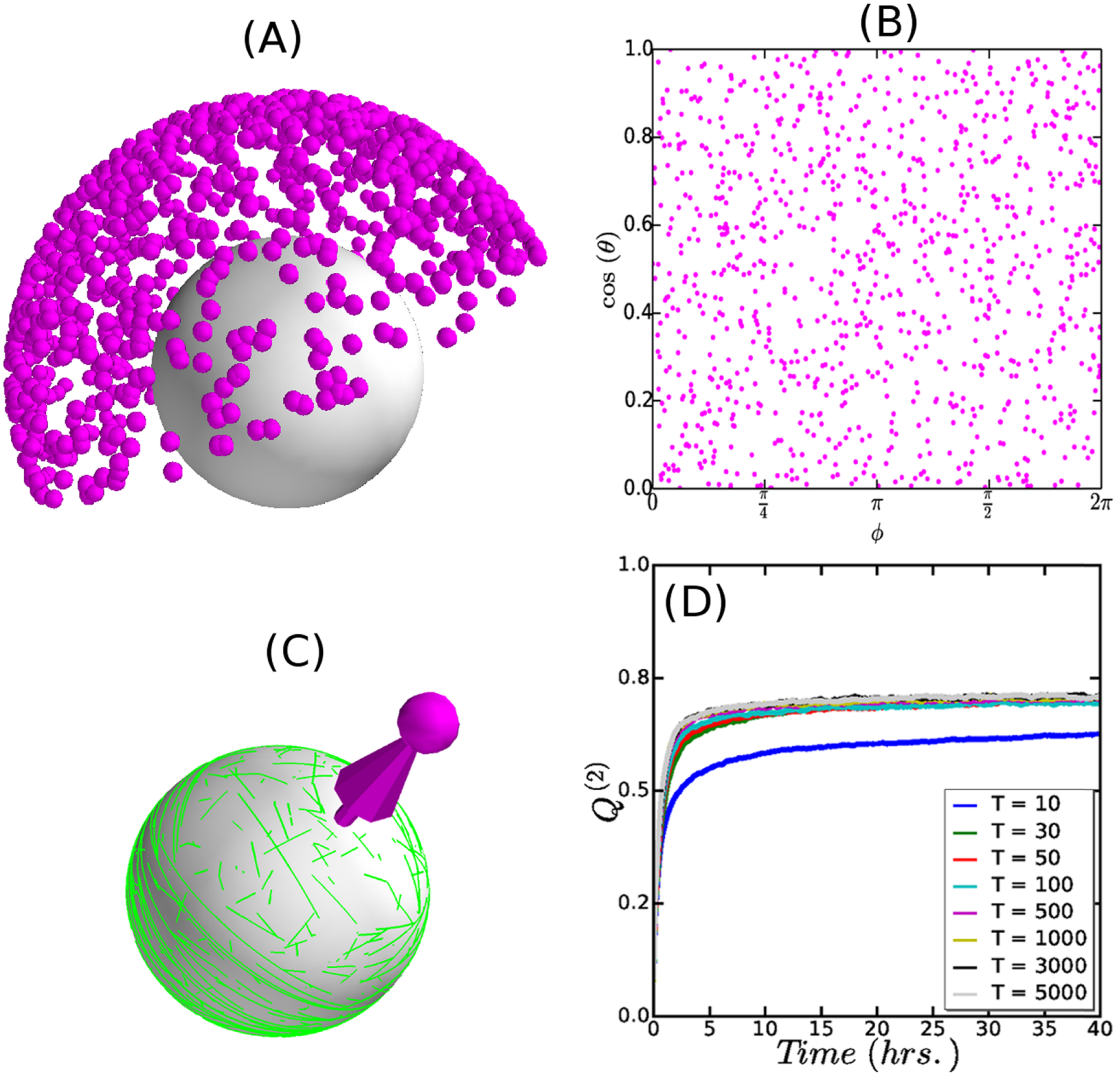
Effect of triangulation on MT array formation. (A) Simulated orientation of MT array on a spherical surface. Simulations were performed for ≈ 1000 independent realizations of the stochastic MT dynamics. (B) Mapping the angular components (*θ*, *ϕ*) of the spherical coordinates of the $\hat{\Omega }$ tips to the equivalent 2D plane (*x*, *y*) (see S1 File, Eq. SI.18). (C) A simulation snapshot of the MT array associated to the specific $\hat{\Omega }$ tip. (D) A comparison in the time evolution of the degree of MT order (*Q*^(2)^) for the different triangulations, reflecting robust reproducibility in the degree of MT order for *T* ≥ 30.

We triangulated a spherical surface with numbers of triangles (*T*) ranging from *T* = 10–5000, using four different triangulation algorithms. On each of these geometries we ran ≈ 1000 independent realizations of the stochastic simulation. Generically, the steady state achieved in these simulations is a diffuse equatorial band of ordered MTs. To test for isotropicity, we considered the distribution of the normalized vectorial order parameter $\hat{\Omega }$. This showed that for *T* > 100 the isotropicity is already satisfactory (see S1 File, Sec. SI.7). We illustrate this in Fig 11(A)–11(C) with the results for *T* = 5000, which is the order of magnitude of *T* obtained by the meshing of the confocal microscope images of *Arabidopsis thaliana* early embryonic cell. Looking at the steady values of the scalar order parameter *Q*^(2)^, we found that, except for the smallest value of *T* which was 10, the results for the time to reach steady state as well as the steady values of *Q*^(2)^ were largely independent of *T*, as illustrated in Fig 11(D). Moreover, these results did not depend on the specific triangulation algorithm used (For details please refer to S1 File, Sec. SI.7).

#### Simulation on cubic surface

**Fig 12:**
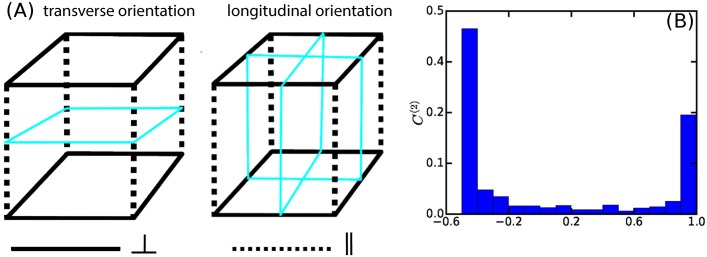
Simulation of MT dynamics on the cubic surface with uniform edge-catastrophe. (A) Schematic diagram of transverse and longitudinal orientations. The transverse orientation is represented as the horizontal planes with boundary edges in solid cyan color and the associated transverse cube edges are flagged by solid black line. The longitudinal orientation is represented as the vertical planes with boundary edges in solid cyan color and the associated longitudinal cube edges are are flagged by dashed black line. (B) Distribution of *C*^(2)^ values for *P*_⊥_ = *P*_∥_ = 0.26, which is a bimodal distribution with peaks at *C*^(2)^ ≈ 1 and *C*^(2)^ ≈ −0.5, obtained from ≈ 1000 independent simulation realizations. In these simulations we used an infinite tubulin pool and the same value of *G* as described in [50].

**Fig 13:**
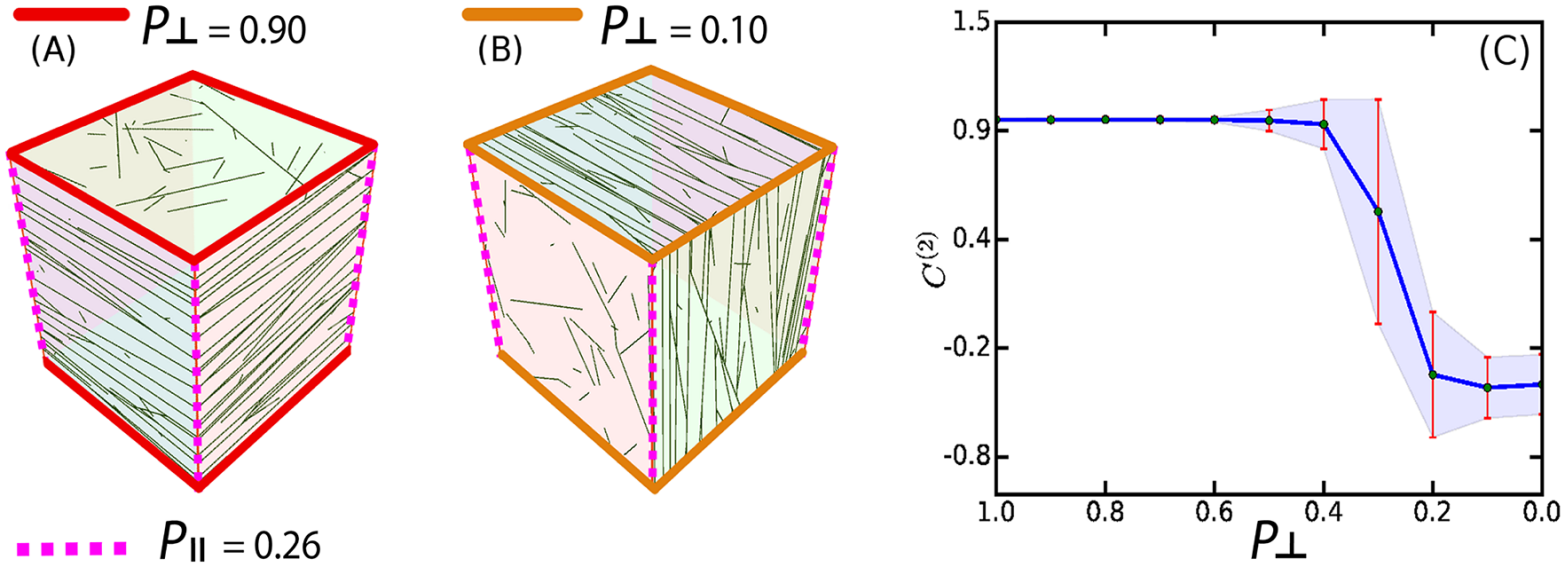
Simulation of MT dynamics on the cubic surface for fixed value of *P*_∥_ = 0.26. Simulated orientation of the MT array for: (A) *P*_⊥_ = 0.9 and (B) *P*_⊥_ = 0.1. (C) Gradual increase in the probability of switching of the MT array orientation from transverse (*C*^(2)^ ≈ 1) to longitudinal (*C*^(2)^ ≈ −0.5). Each point in the plot was an average of ≈ 1000 independent simulations. Parameter values used for this simulations are as described in [50].

To test the ability of our simulation framework to reproduce existing MT simulation results, we considered a cubical geometry. In the original simulation reported in [50], the cube was built up out of 6 square planar domains. Here, we triangulated the cube by subdividing each face along the diagonals into 4 isosceles triangles. The cube-edges between the faces were classified into two groups: the ones bordering the top and bottom faces are denoted as transverse and the ones along the sides as longitudinal as shown in Fig 12(A). Assuming a virtual organ axis to run vertically, then in keeping with standard nomenclature the transverse orientation would be denoted as transverse anticlinal, while the two longitudinal orientations would be the radial anticlinal and the periclinal orientation respectively, with the choice between the latter two dependent on the orientation on the lateral surface of the organ. *Edge-catastrophe* probabilities were associated to the cube-edges, denoted by *P*_⊥_ and *P*_∥_ for the transverse and longitudinal edges respectively. Following [50], we adopt the following orientational order parameter $C^{\left(2\right)}=\Omega_{z}-\frac{1}{2}\sqrt{\Omega_{x}^{2}+\Omega_{y}^{2}}$, where the *z*-axis is chosen perpendicular to the top and bottom face. A value of *C*^(2)^ = 1 corresponds to prefect transverse orientation of the array, while *C*^(2)^ = −1/2 signals a longitudinal orientation. The intermediate value *C*^(2)^ = 0 corresponds to a mixed state with an equal probability for each of the three principal ordering orientations. To obtain the unbiased state, we first considered *P*_⊥_ = *P*_∥_ = 0.26, the value reported in [34] for the *edge-catastrophe* probability at the periclinal edges in *Arabidopsis thaliana* root epidermis cells. As Fig 12(B), we find a distribution of *C*^(2)^ values, with roughly 2/3 with a value of *C*^(2)^ ≈ 0.5 corresponding to the two equivalent longitudinal orientations, and 1/3 with *C*^(2)^ ≈ 1, corresponding to the transverse orientation. We then keep *P*_∥_ = 0.26 fixed, and scan *P*_⊥_ in the range 0–1 from high to low values. We observed (see Fig 13) that initially the system remains locked into the transverse orientation characterized by *C*^(2)^ ≈ 1. When the value of *P*_⊥_ become comparable to *P*_∥_ (0.2 ⪅ *P*_⊥_ ⪅ 0.3) the system enters a transition regime in which it can stabilize to either the transverse or the longitudinal state with relative frequencies dependent on the precise value of *P*_⊥_. Finally, for *P*_⊥_ ⪅ 0.1, only the longitudinal orientation (with equal probability in both of the possible orientations), was observed, i.e. a state flipped by a complete 90° degree angle from the original transverse orientation. The results are fully consistent with those reported previously [50], including the occurrence of an additional set of diagonal MT orientations when *edge-catastrophes* are absent (see S1 File, Sec. SI.8).

### Probing the influence of cell shape on MT order

We are now in a position to use our framework to study the role of cell shape on MT array formation. We first do this in a geometrically simple setting, which allows us to systematically disentangle the role the various shape-related factors involved. Then we turn real cell shapes, provided by the leaf pavement cells of *Arabidopsis thaliana*, *Hedera helix* and *Nicotiana benthamiana*.

#### Interplay between shape effects and stability rules

**Fig 14:**
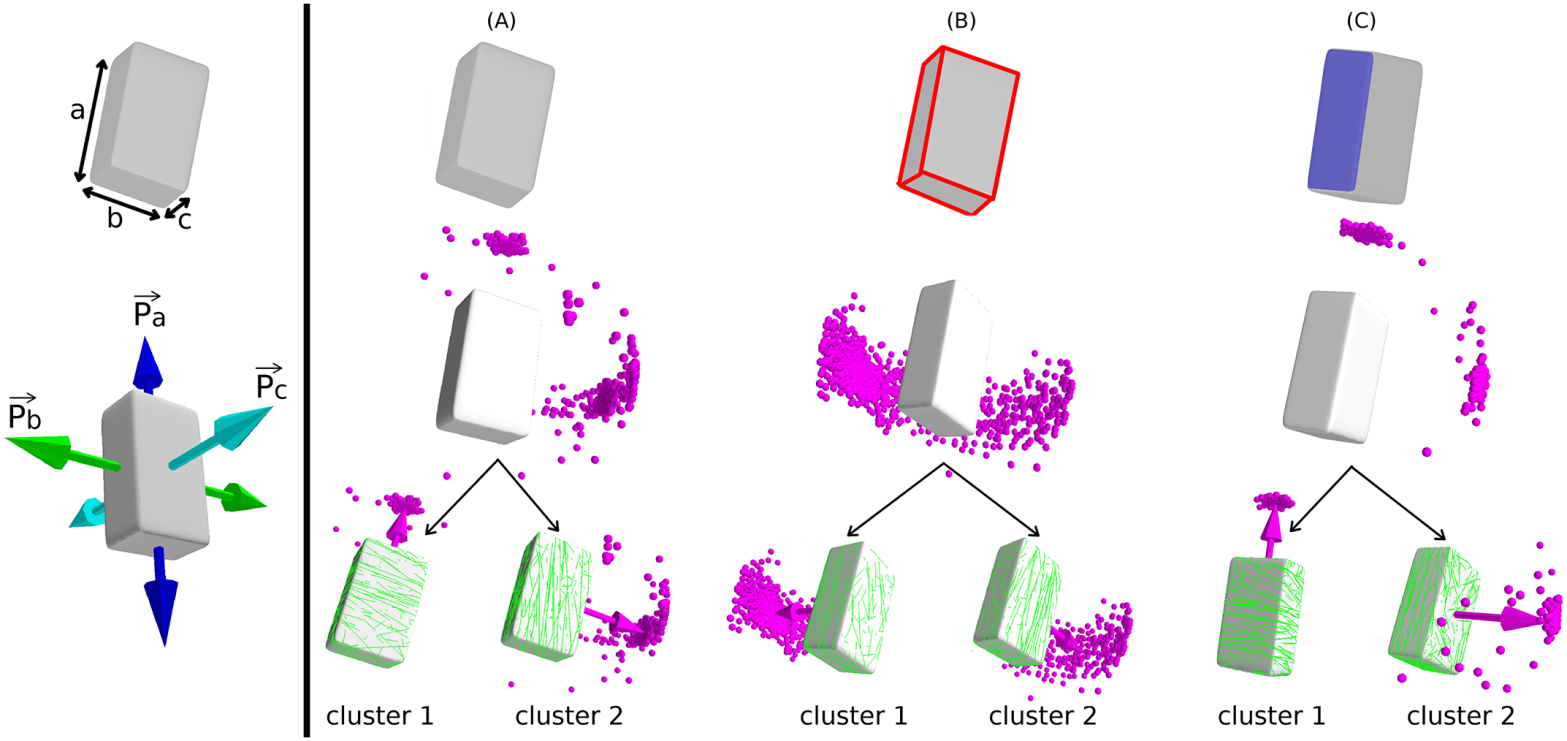
Simulation of MT dynamics on the rectangular parallelepiped surface. A Schematic representation of a rectangular parallelepiped and the symmetry direction ${\vec{p}}_{a}$,${\vec{p}}_{b}$ and ${\vec{p}}_{c}$ parallel to the edges *a*, *b* and *c* respectively (left panel). MTs were simulated by considering three different regulatory effects in the dynamics of MT: (A) default shape, which resulted in two clusters (cluster 1: ≈ 52% [519/991] around ${\vec{p}}_{a}$, cluster 2: ≈ 46% [452/991] around ${\vec{p}}_{b}$) of MT array orientation, (B) implementing *edge-catastrophe*, which resulted in two clusters (cluster 1: ≈ 28% [276/992] around ${\vec{p}}_{b}$, cluster 2: ≈ 71% [706/992] around ${\vec{p}}_{c}$), and (C) enhanced MT stabilization at a face of largest area, which resulted in two different clusters (cluster 1: ≈ 77% [762/995] around ${\vec{p}}_{a}$, cluster 2: ≈ 19% [186/995] around ${\vec{p}}_{c}$) of array orientations.

As the example of the cubical surface discussed above shows, shape by itself already has an impact on MT order and array orientation, essentially selecting a finite number of possible stable array orientations. Adding *edge-catastrophes* can then serve to uniquely stabilize one of these possibilities. There is, however, a third factor that could play a role, namely differences in MT dynamics localized to specific cell-faces. The possibility of cell-face dependent MT dynamics has been discussed in the context of array organisation due to environmental cues such as light [51] and hormone stimulation [52]. Such differences could potentially also derive from developmental differences between the cell-faces, e.g. the difference between faces created by division versus expanding cell-faces, or maturation effects due to “ageing” of the associated face. Here we assess the interplay between these three factors in a non-trivial, yet simple cell shape. We triangulated a rectangular parallelepiped of dimensions *a* = 13 *μm*, *b* = 7.5 *μm* and *c* = 5 *μm*, subsequently rounding its edges using *meshLab* [39]. These dimensions, and the resultant cell surface area, were chosen to provide a highly stylized version of an early stage *Arabidopsis thaliana* embryonic cell. The MT dynamical parameters were chosen from the working domain of the simulation to yield a steady state value of $\bar{l}=4.5\;\mu m$, ensuring that MTs are sensitive both to edge and shape effects. To discuss the possible orientations of the MT array, we introduce three unit vectors ${\hat{p}}_{a}$, ${\hat{p}}_{b}$ and ${\hat{p}}_{c}$ parallel to corresponding edges (see Fig 14, left panel). The results for the observed CA orientations were classified using the standard Python clustering package fcluster, which allows identification of a pre-defined number of clusters from a given set of points based on the relative distances between the points. The relevant number of clusters is obtained by visual inspection of the results.

In the default case, i.e. without any additional effects, average value of the global scalar order parameter was *Q*^(2)^ ≈ 0.7 with 52% of all realizations the vector order parameter aligns along the ${\hat{p}}_{a}$ direction, in 46% along the ${\hat{p}}_{b}$ direction, and the remaining few percent in spurious directions, but notably none along the ${\hat{p}}_{c}$ direction (see Fig 14(A), right panel). To interpret these observations, we first remark that MT arrays tend to evolve to a state that minimizes the number of inter-MT collisions, the hallmark of the ‘survival of the aligned’ mechanism. On a closed surface this will favor directions in which non-intersecting closed paths can exist on the surface. Such a state requires the orientations of individual MTs to be correlated spatially along the preferred directions. Since the length of individual MTs is typically smaller than the lengths of the closed paths, this correlated state is more readily maintained on shorter paths, which explains the propensity to select directions corresponding to geodetic paths, already observed in the case of the spherical and cubical shapes discussed above. This argument immediately predicts that a parallel MT array perpendicular to ${\hat{p}}_{a}$, which covers the shortest circumference *C*_*a*_ = 2(*b* + *c*) = 12.5*μm*, should be most favoured, followed by the orientation ${\hat{p}}_{b}$, with *C*_*b*_ = 2(*a* + *c*) = 18*μm*, whereas the least favored direction ${\hat{p}}_{c}$ with *C*_*c*_ = 2(*a* + *b*) = 20.5 *μm* apparently is no longer stabilized.

Implementing *edge-catastrophes* as described in S1 File Sec. SI.2 with *edge-catastrophe* multiplier *E*_*cat*_ ≥ 0.5 to all the edges of the rectangular parallelepiped completely reverses this tendency (see Fig 14(B), right panel). Directions in which MTs would on average cross more catastrophe-inducing edges over their length become unfavorable. In this case the orientation ${\hat{p}}_{c}$ with average distance between edge crossings $d_{c}=\frac{1}{2}\left(a+b\right)=5.125\mu m$ is most likely (71%), followed by ${\hat{p}}_{b}$ with $d_{b}=\frac{1}{2}\left(a+c\right)=4.5\mu m$ with 28%, whereas now ${\hat{p}}_{a}$ with $d_{a}=\frac{1}{2}\left(b+c\right)=3.125\mu m$ did not occur at all. The average value of the global scalar order parameter was *Q*^(2)^ ≈ 0.6 for *E*_*cat*_ = 0.5 and further decreased for higher values of *E*_*cat*_ without much effect on the distribution of MT array orientations.

Finally, we considered the role of face stabilization on MT dynamics. To that end we “protected” the faces with the surface area (*a* × *c*) and increased the MT *spontaneous-catastrophe* rate *r*_*c*_ on all other faces such that the average length of the MTs on those faces decreases to $\bar{l}=3.5\mu m$ (see Fig 14(C), right panel). This obviously favors arrays which pass over the face with edges (*a*, *c*). Indeed the results now show an uneven split between the orientations given by ${\hat{p}}_{a}$ (77%) and ${\hat{p}}_{c}$ (19%) with average value of the global scalar order parameter, *Q*^(2)^ ≈ 0.8. In this case the direction ${\hat{p}}_{b}$ is explicitly suppressed as it would require MTs to pass only through faces with lower stability.

Taken together these results show that both *edge-catastrophe* and face stability can independently exert a degree of control over the global MT array state.

#### MT simulations on realistic plant cell shapes

**Fig 15:**
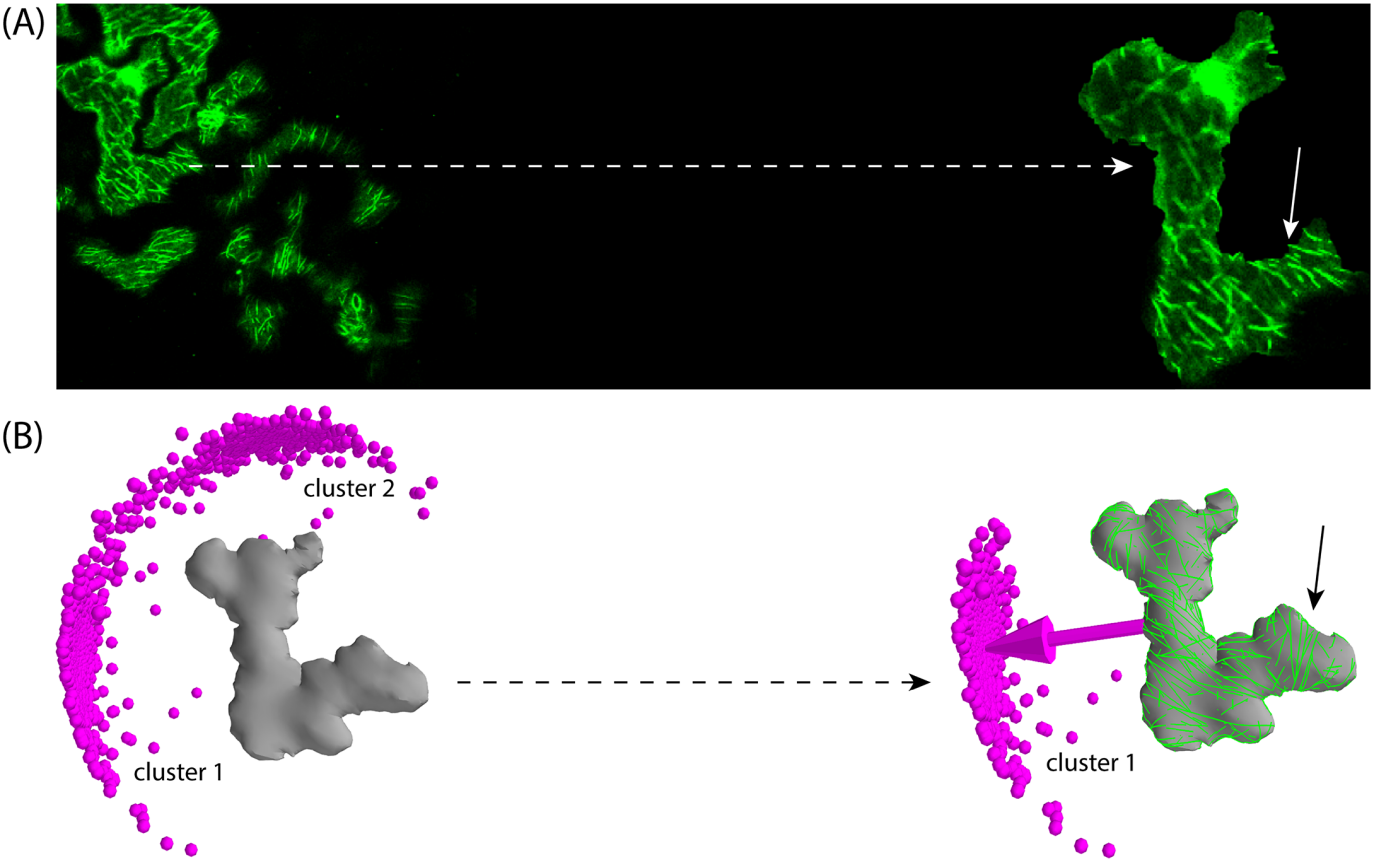
Comparison of experimental to simulated orientation of MT array on the surface (cortex) of leaf pavement cell of *Arabidopsis thaliana*. (A) MAP65-decorated MT array in *Arabidopsis thaliana* leaf pavement cell. One cell with multiple protrusions and an ordered array of MTs is highlighted by the dashed arrow.(B) Simulated orientation of MT arrays on the surface of the same cell. Two clusters of MT array orientation were formed (cluster 1: ≈ 56% [559/992] and cluster 2: ≈ 44% [433/992]). MT array pattern associated to the average orientation of the dominant cluster, i.e. cluster 1, correlated well with the experimentally captured MT array pattern shown in panel (A). An instance of experimental and simulated formation of MT bands around the neck of protrusions is indicated by solid arrows.

Although of varying degree of regularity (see Fig 1), some plant cells have highly non-trivial shapes. A case in point is the shapes of the leaf pavement cells of flowering plants such as *Arabidopsis thaliana*, *Nicotiana benthamiana* and *Hedera helix*, which have multiple highly irregular protrusions. As such they provide an ideal test case for our framework. We first segmented confocal images of such cells, produced a triangulated approximation to their shapes, followed by manual tagging of cell-edges and cell-faces when applicable. Generally, these cells are bigger than the embryonic cells, however we rescaled their surface area to the average surface area of the embryonic cells, without affecting their shape. This allows us to use simulation parameters from the previously defined working domain, ensuring formation of an ordered MT array in the same time scale. We simulated the MT dynamics on the resultant triangulated networks of the cells under default conditions. As a case study, we first visualized the MT array pattern on the inner membrane cortex of *Arabidopsis thaliana* leaf pavement cell (see Fig 15(A)). MAP65 is known to be a well studied protein family that participates in the polymerization and bundling of MTs, therefore we used 35S::MAP65-GFP lines to visualize the cortical MTs [53]. Our experimental observation showed instances of MT band formation around the necks of protrusions, which has already been reported [54]. Our simulation on the respective cell shape resulted in a sparse distribution of MT array orientations with two major populations (cluster 1: ≈ 56% and cluster 2: ≈ 44%) of MT array orientations (see Fig 15(B)). Interestingly, the average MT array orientation of the maximally populated cluster, i.e. cluster 1, corresponded to our experimentally captured MT array pattern. Our experimentally captured MT array pattern (Fig 15(A) right panel) and a simulation snapshot of MT arrays (Fig 15(B) right panel) also revealed an instance of formation of MT bands around the necks of protrusions, highlighted by solid arrow in Fig 15.

**Fig 16:**
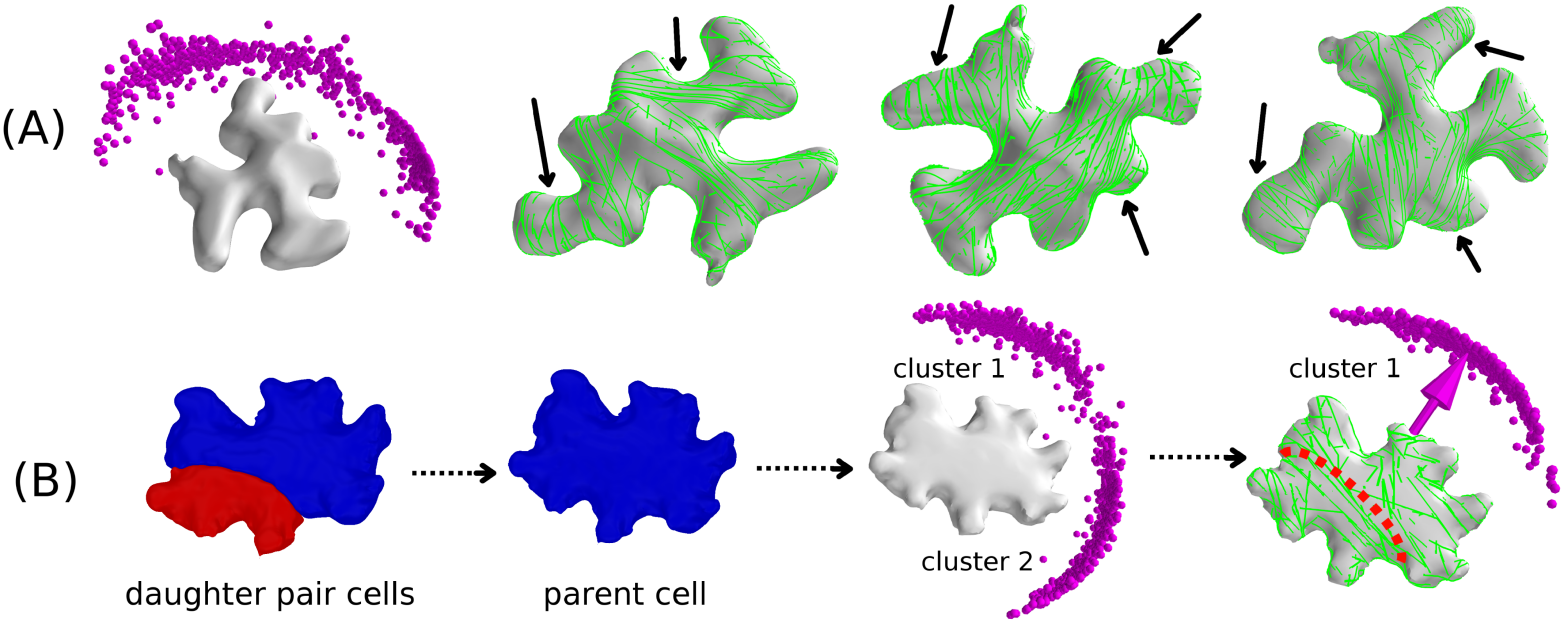
Simulated orientation of MT arrays on the surface of leaf pavement cell shapes of *Nicotiana benthamiana* and *Hedera helix*. Simulations were performed by considering basic MT-MT interactions on default cell shapes: (A) Simulated orientation of MT arrays on the leaf pavement cell of *Nicotiana benthamiana*. Resulting orientation was almost a random distribution, which was restricted on the semi-2D plane of the cell shape and reflected formation of local MT arrays around the various irregular protrusions (three different instances are shown by arrows). Various snapshots reveal a significant degree of local order, which is also reflected in the value of the global scalar order parameter *Q*^(2)^ ≈ 0.45. (B) A parent leaf pavement cell shape of *Hedera helix* was recreated by merging the daughter pair of cells, then MT dynamics was simulated on the default shape which resulted in two clusters of MT array orientation with almost identical probabilities (cluster 1: ≈ 51% [509/991] and cluster 2: ≈ 49% [482/991]). Average MT array orientation associated with the cluster 1 matched with the corresponding orientation of the division plane that created the daughter pair of cells.

In the leaf pavement cell of *Nicotiana benthamiana*, MT bands around the necks of protrusions have also been observed [55–59], which we further confirmed by using 35S::TUB-mCHERRY lines to visualize MTs on the inner membrane cortex of such cell type (see S1 File, Sec. SI.9). This gave us an excellent opportunity to further explore the influence of cell shapes on the formation of such MT bands through simulating MT dynamics on these cell shapes. Our simulation resulted into almost random distributions of the global ordering orientation, restricted, however, to the semi-2D plane of the shape itself. Local order included in several cases band formation around the necks of protrusions (see Fig 16(A)). Further, evidence was presented that in non-trivial shape such as leaf pavemet cells, actin is involved in localising these structures [54]. The requirement for actin to constrain MT bands around the necks is consistent with our result that MT dynamics influenced by geometry alone cannot exclusively generate these “neck” configurations. As the snapshots show, stochastic differences between individual realisations of steady state configurations lead to varying distributions of banded structures over the shape, which also explains the essentially random global array orientations in our simulations. In addition, we can now look for a possible correlation between a robust prediction of global orientation of the MT array and the subsequent orientation of division plane [60, 61], by simulating MT dynamics on the triangulated network of a reconstructed parental *Hedera helix* leaf pavement cell. We used *morphographX* [38] to recreate such a “parent cell”, by merging the corresponding daughter cell pair. The simulations revealed two distinct possibilities for average global ordering orientation as shown in Fig 16(B), with very similar frequency of occurrence: Cluster 1 ≈ 51% and cluster 2 ≈ 49%. Cluster 1 is slightly more compact, indicating a smaller standard deviation in the corresponding orientation. Consistent with the experimental observation, the orientation of cluster 1 corresponds to the actual orientation of the division plane that generated the cell pair. This shows that, at the very least, cell geometry alone, in the absence of additional controlling mechanisms, can predispose array orientation towards selecting division planes. Note that our simulations also appear to rule out the possibility of out-of-plane array orientation, consistent with the fact that periclinal divisions (parallel to the leaf surface) are extremely rare in leaf epidermis cells (maize [62], tobacco [63]).

**Fig 17:**
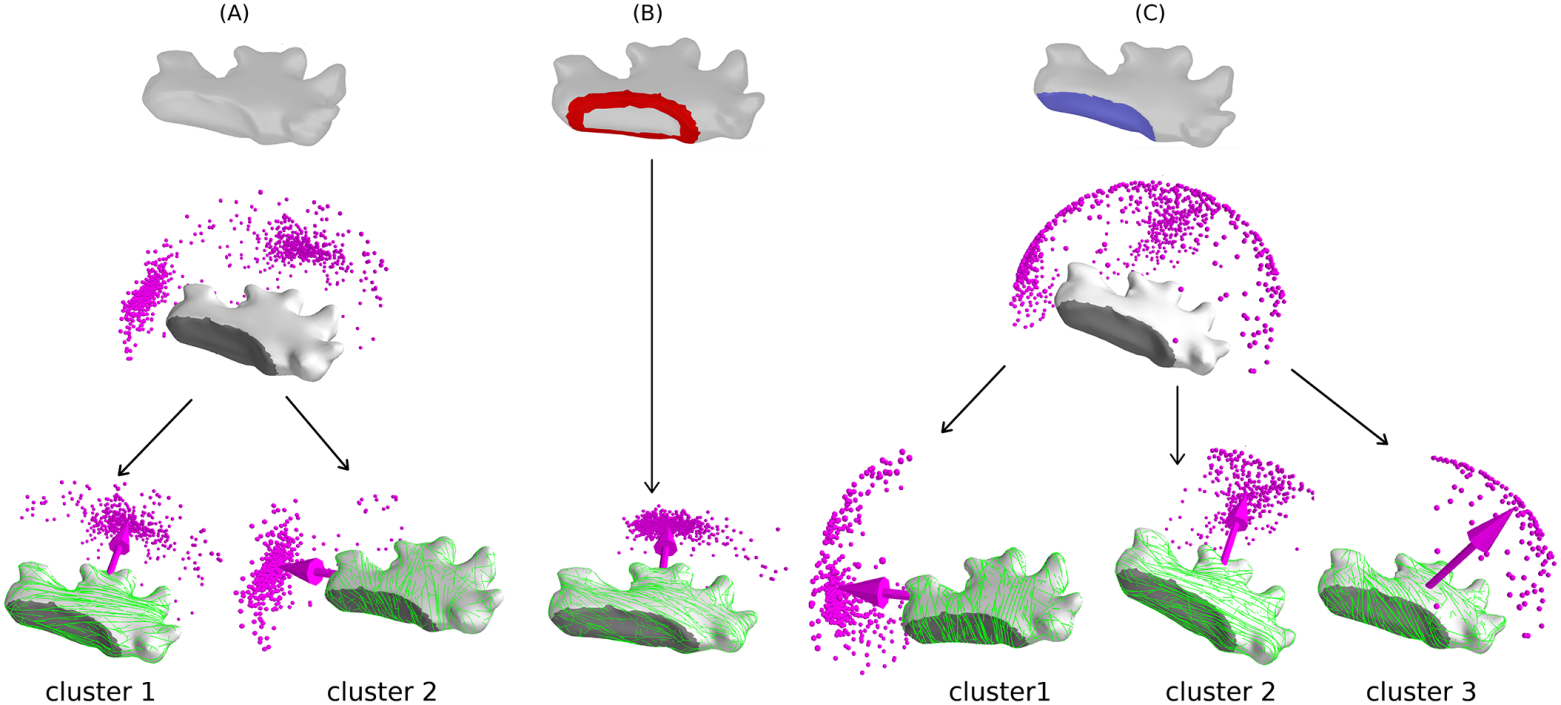
Simulated orientation of MT arrays on the surface of the recent generation of *Hedera helix* leaf pavement cell shape. (A) Simulation on default shape, which resulted in two clusters of MT array orientation with almost identical probabilities (cluster 1: ≈ 47% [522/993] and cluster 2: ≈ 53% [471/993],(B) Simulation with *edge-catastrophe*, which resulted in a single cluster of MT array orientation. Edge of the cell is coloured red to indicate that in simulation, the MTs were subjected to *edge-catastrophes*, and (C) Simulation with enhanced MT stabilization at the developmentally new cell-face (here the basal face), which resulted in the formation three clusters of MT array orientation. Majority of the orientations were distributed in two nearly identical clusters (cluster 1: ≈ 37% [370/993] and cluster 2: ≈ 49% [484/993]), reflecting persistence of the default shape influence. Additionally, a small fraction of the MT array orientation formed a third cluster (cluster 3: ≈ 14% [139/993]), which depicts the effect of enhanced MT stabilization at the developmentally new cell-face.

Finally, we applied our simulation framework on a most recent generation of *Hedera helix* pavement cell, i.e a daughter cell of a just divided cell. These cells contain a cell-edge and two developmentally distinct cell-faces, thus providing a suitable realistic sample to explore the possible consequences of *edge-catastrophe* and MT stabilization on the MT array orientation. Simulations on default shape resulted in two possibilities (cluster 1 ≈ 47% and cluster 2 ≈ 53%) of average global ordering orientation (see Fig 17(A)), however incorporation of *edge-catastrophes* to the circumference of the basal cell-face uniquely selects only a single average orientation of MT array (see Fig 17(B)). This again highlights the ability of cell intrinsic features, such as the sharp cell-edges created by a preceding division plane, to uniquely determine array orientation, and therefore potentially the subsequent division plane. However, it is conceivable that for developmental purposes the cell would require additional control, beyond the auto-regulatory effects of cell geometry alone. We argue that one such form of control could arise from the differential properties of newly created cell-faces. These faces are developmentally distinct through their genesis, which could also influence their local biochemical state. Another possible mechanism could involve polarization effects induced by localization of Auxin transporters, such as the PIN family of protein [64, 65]. In either case the local biochemical state could impact MT dynamics, such as to impact their stability. Here, we limit ourselves to local enhancement of MT stability in the basal cell-face, achieved by 2-fold increase of the local *spontaneous-catastrophe* rate *r*_*c*_ in the face other than the basal face. This parameterization decreased the $\bar{l}$ to 3.5 *μm* in that face, where the $\bar{l}$ in the basal face was 4.5 *μm*. As Fig 17(C) shows, we again found two major orientations of MT arrays as observed in the default shape simulation, but now with slightly decreased propensities (cluster 1 ≈ 37% and cluster 2 ≈ 49%). However, a new orientation appears in ≈ 14% (cluster 3) of realizations, in an unexpected tilted direction. This shows that face stability indeed provides an additional input for independent control, that is able to (at least partially) override the geometrical mechanisms based on avoidance of self-intersections.

## Discussion

The modelling framework described in this article allows the simulation of cortical MT dynamics on triangulated approximations of essentially arbitrary 3D shapes, allowing for the first time to address the interplay between cell shape and MT array organisation on realistic cell shapes, which recent work has highlighted as being important [4]. This enables the systematic exploration of the predictions that the current consensus hypothesis on CA formation makes in a biologically realistic context. A diverse set of precise predictions will facilitate the indispensable experimental testing of the hypothesis, and aid the discovery of potentially important factors hitherto not taken into account.

A crucial ingredient of the system is the ability to deal with localized variations in the MT dynamics. The well established propensity of MTs to undergo catastrophes when crossing high curvature regions between distinct cell-faces is readily implemented, by identifying—for now by hand, but potentially automatically in the future—the regions involved. The second, is the, for now putative, yet biologically reasonable assumption of distinct levels of MT stability on different cell-faces, due to the difference in their development, e.g. newly created faces due to cell division versus cell-faces that expand through growth. The results, both on the simplified cuboidal geometry and the complex leaf pavement cells of *Arabidopsis thaliana*, *Nicotiana benthamiana* and *Hedera helix*, show that cell geometry alone already strongly restricts the possible array orientations. In the leaf pavement cells it can, moreover, predict the occurrence of non-trivial features such as band formation around neck-like protrusions. Both *edge-catastrophe* and face stability effects can then serve to fine-tune, or in some cases override, the spectrum of array orientations already limited by the cell geometry per se. Heuristically, the interplay between these different effects can be summarized as follows: (i) geometry favors MT trajectories that collectively form closed geodetic paths on the surface (ii) *edge-catastrophes* will select those closed paths that have fewest edge crossings, while (iii) enhanced face stability will select those paths that maximally intersect the face selected to have enhanced MT stability. It is precisely the balance between the latter two effects, which depending on the specific geometry can be either synergistic or antagonistic, that could provide a mechanism of cellular control over array orientation. As a rule, interphase array orientation parallels the orientation of the pre-prophase band, which in turn is a predictor for the location and orientation of the future division plane [60, 61]. This suggests that insights in MT organisation from our simulations could be used to address long-standing issues in plant morphogenesis, which to a large degree is governed by the interplay between division plane orientation and cell growth. A first hint in that direction comes from our result on prediction of the possible division planes in merged daughter cells of the leaf pavement cell of *Hedera helix*.

Here we did not dwell on additional relevant factors such as the role of anisotropic MT bound nucleation [66, 67] and MT severing by Katanin [68]. Both these effects, however, have already been implemented in the underlying MT interaction algorithms, and have been reported on elsewhere [69, 70].

Recent studies have provided evidence that MT dynamics is sensitive to the distribution of mechanical stress in the cell wall [71, 72]. Our modelling framework has the ability to encode domain specific parametrization of MT dynamics. Therefore, once the precise influence of mechanical stress on the dynamics of MT is known, our modelling framework should allow the effects of a static stress distribution to be incorporated. In the current form, however, our modelling framework relies on the static template of a triangulated surface. It can therefore arguably only straightforwardly be applied to non- or slowly growing plant cells, for example those in early embryo development, where cell shape evolve slowly compared to e.g. division time.

In the context of growing cells, such as anisotropically growing root epidermal cells, the feedback mechanism between cortical MT dynamics, cell shape evolution and growth induced anisotropic stress needs to be considered. It will be a challenging, but potentially feasible task to extend our framework to simulate MT dynamics on growing cells as well, where cell growth e.g. is simulated by using the Finite Element Method [73–75].

### Experimental procedure

*Arabidopsis thaliana* and *Nicotiana benthamiana* plants were grown as described in [76]. Leaves from these plant species were harvested and incubated in Renaissance staining as described in [77], samples were then mounted in water to visualized their cell shape. 35S::MAP65-GFP expression in *Arabidopsis thaliana* leaves was analysed using 3 days old seedlings and 4 weeks old plants respectively. Expression assays in *Nicotiana benthamiana* leaves were performed using 4 weeks old plants. Agrobacterium containing 35S::TUB-mCHERRY were infiltrated as described in [78].

*Arabidopsis thaliana* leaves were stained with Propidium idodide (10*μg*/ml) and imaged using a C- Apochromat 40 X/1.20 W Korr water immersion objective of a LSM 880 airyscan Zeiss confocal microscopes. GFP was excited using an argon laser 488nm, and fluorescence emission was detected from 500 to 540 nm. Renaissance staining for *Nicotiana benthamiana* leaves was detected at 415–440 nm excitation and a 405 nm beam splitter. For 35S::TUB-mCHERRY localization in *Nicotiana benthamiana*, infiltrated leaves were mounted in water and mCHERRY was detected with 543 nm excitation and 488/543/633 beam splitter.

## Supporting information

S1 Fig Definition of edge angle. Left panel: Faces *F*_1_ (blue colour)and *F*_2_ (green colour), meet along the dotted line, (B) Using this dotted line as reference, an edge *E* (red colour) is detected, which is composed of multiple triangles belonging to either *F*_1_ or *F*_2_. Right panel: Triangles with edge color (*e*_*c*_; red) and one of the face colour (*f*_1_;blue or *f*_2_;green), are identified via the respective face colour only.
(TIF)

S2 FigDefinition of bending angle.Schematic diagram of MT bending angle calculations at an edge. Left panel: (A) Edge angle $\theta_{E}^{avg}$, (B) *Incidence angle* *θ*_*i*_: the angle between the direction of MT growth ${\hat{m}}_{P}$ along the trajectory M and the tangent ${\hat{e}}_{P}$ to the curve E of maximal curvature between the adjacent faces at the crossing point *P*. (C) Bending angle *θ*_*b*_.(TIF)

S3 FigImplementation of edge-catastrophe.The propensity for *edge-catastrophe* in MT dynamics is determined through local bending of MTs through a set of triangle pairs (*T*_1_, *T*_2_), which belong to an edge (*k*). ${\hat{m}}_{\left(k\right)}$ is the growth direction of a MT passing from $T_{1}^{\left(k\right)}$ to $T_{2}^{\left(k\right)}$ through their shared edge *k* and ${\hat{t}}_{\left(k\right)}$ is a unit vector along this edge.(TIF)

S4 FigImplementation of differential MT stabilization.When a MT passes from a triangle *T*_*i*_ of a face *F*_1_ to a triangle *T*_*j*_ of another face *F*_2_, we update its *spontaneous catastrophe* from $r_{c}^{F_{1}}\to r_{c}^{F_{2}}$.(TIF)

S5 FigTubulin pool size effects.Comparison of MT ordering under an infinite tubulin pool and a finite tubulin pool and comparison between simulated ($\bar{l}$) and theoretical (*l*_*avg*_) average MT length. (A) Time evolution of *Q*^(2)^ for: (1) *G* with infinite tubulin pool (*ρ*_*tub*_ = ∞ *μm*^−1^), and (2) *G*_*eff*_ with finite tubulin pool (*ρ*_*tub*_ = 10 *μm*^−1^). Due to presence of finite tubulin pool effect, calculation of *G* by using modified value of MT plus-end growth speed resulted in a modified value from *G* ≈ −0.005 to *G*_*eff*_ ≈ −0.05. (B) For different values of *l*_0_, variation of simulated MT average length $\bar{l}$ which includes interaction effects, with respect to *l*_*avg*_ which excludes any interaction effects.(TIF)

S6 FigHomogeneity of orientations on a sphere as a function of triangle number.(A) Distribution of $\hat{\Omega }$ on the surface of a sphere, triangulated by different numbers of triangles (*T* = 10, 30, 50, 100, 1000, 3000, 5000). (B) Distribution of $\hat{\Omega }$ on the surface of a sphere, triangulated by different algorithms (*T* = *I*, *II*, *III*, *IV*) while keeping number of triangles fixed at *T* = 5000. (C) The Chi-squared test for homogeneity in the distribution of $\hat{\Omega }$ tips for each case of triangulation. With the increasing number of triangles, the corresponding distribution of $\hat{\Omega }$ tips becomes more homogeneous.(TIF)

S7 Fig Array orientations on a cubical cell. Simulated orientation of MT arrays on default cube surface with side length *L* = 15*μm*: (A) Formation of MT arrays along the nine most favoured closed geodetic paths. (B) Additionally, we also found four less-favoured paths of MT array formation, which are composed completely of diagonal paths from different faces.
(TIF)

S8 Fig Experimental observation of MT array in a *Nicotiana benthamiana* cell. MT array pattern on the inner membrane cortex of *Nicotiana benthamiana* leaf pavement cell. 35S::TUB-mCHERRY lines were used to visualize the cortical MTs and ordered arrays of MTs are highlighted by the dashed arrows.
(TIF)

S1 Table Simulation parameters. Overview of the MT dynamics parameters and variables with their default values (if applicable). For description and sources see S1 File, Sec. SI.5.
(PDF)

S1 File Additional technical details. We provide the details of the definition of edge angle, the implementation of edge-catastrophes and MT stabilization, the definition of the order parameter tensor, the implementation of finite tubulin pool effects, the parametrization of the simulations, and the analysis of the effects of triangulation of the surface.
(PDF)

## References

[1] Sablowski R. Coordination of plant cell growth and division: collective control or mutual agreement? Current Opinion in Plant Biology. 2016;34:54–60. 34 Cell biology 2016. doi: 10.1016/j.pbi.2016.09.004 27723536 10.1016/j.pbi.2016.09.004

[2] Ehrhardt DW. Straighten up and fly right: microtubule dynamics and organization of non-centrosomal arrays in higher plants. Curr Opin Cell Biol. 2008;20:107 doi: 10.1016/j.ceb.2007.12.004 18243678 10.1016/j.ceb.2007.12.004

[3] Ehrhardt DW, Shaw SL. Microtubule dynamics and organization in the plant cortical array. Annu Rev Plant Biol. 2006;57:859 doi: 10.1146/annurev.arplant.57.032905.105329 16669785 10.1146/annurev.arplant.57.032905.105329

[4] Gomez JM, Chumakova L, Bulgakova NA, Brown NH. Microtubule organization is determined by the shape of epithelial cells. Nat Com. 2016;7:13172 doi: 10.1038/ncomms13172 10.1038/ncomms13172 PMC5093320 27779189

[5] Paredez AR, Somerville CR, Ehrhardt DW. Visualization of cellulose synthase demonstrates functional association with microtubules. Science. 2006;312:1491 doi: 10.1126/science.1126551 16627697 10.1126/science.1126551

[6] Emons AMC, Höfte H, Mulder BM. Microtubules and cellulose microfibrils: how intimate is their relationship? Trends in Plant Science. 2007;12:279 doi: 10.1016/j.tplants.2007.06.002 17591457 10.1016/j.tplants.2007.06.002

[7] Lindeboom J, Mulder BM, Vos JW, Ketelaar T, Emons AM. Cellulose microfibril deposition: coordinated activity at the plant plasma membrane. J Microsc. 2008;231:192 doi: 10.1111/j.1365-2818.2008.02035.x 18778417 10.1111/j.1365-2818.2008.02035.x

[8] Lloyd CW. Plant Microtubules: Their Role in Growth and Development. John Wiley & Sons, Ltd; 2001.

[9] Hamant O, Heisler MG, Jönsson H, Krupinski P, Uyttewaal M, Bokov P, et al Developmental patterning by mechanical signals in Arabidopsis. Science. 2008;322:1650 doi: 10.1126/science.1165594 19074340 10.1126/science.1165594

[10] Wasteneys GO. Microtubule organization in the green kingdom: chaos or self-order? Journal of Cell Science. 2002;115:1345 11896182 10.1242/jcs.115.7.1345

[11] Mandelkow E, Mandelkow EM. Microtubule structure. Current opinion in Structural Biology. 1994;4:171 doi: 10.1016/S0959-440X(94)90305-0

[12] Rothwell SW, Grasser WA, Murphy DB. Direct Observation of Microtubule Treadmilling by Electron Microscopy. The Journal of Cell Biology. 1985;101:1637 doi: 10.1083/jcb.101.5.1637 4055889 10.1083/jcb.101.5.1637 PMC2113982

[13] Panda D, Miller HP, Wilson L. Rapid treadmilling of brain microtubules free of microtubule-associated proteins in vitro and its suppression by tau. Proc Natl Acad Sci USA. 1999;96:12459 doi: 10.1073/pnas.96.22.12459 10535944 10.1073/pnas.96.22.12459 PMC22948

[14] Grego S, Cantillana V, Salmon ED. Microtubule Treadmilling in Vitro Investigated by Fluorescence Speckle and Confocal Microscopy. Biophysical Journal. 2001;81:66 doi: 10.1016/S0006-3495(01)75680-9 11423395 10.1016/S0006-3495(01)75680-9 PMC1301492

[15] Rodionov VI, Borisy GG. Microtubule Treadmilling in Vivo. Science. 1997;275:215 doi: 10.1126/science.275.5297.215 8985015 10.1126/science.275.5297.215

[16] Schmit AC. A centrosomal microtubule nucleation in higher plants. Int Rev Cytol. 2002;220:257 doi: 10.1016/S0074-7696(02)20008-X 12224551 10.1016/s0074-7696(02)20008-x

[17] Shaw SL, Kamyar R, Ehrhardt DW. Sustained microtubule treadmilling in Arabidopsis cortical arrays. Science. 2003;300:1715 doi: 10.1126/science.1083529 12714675 10.1126/science.1083529

[18] Vos JW, Sieberer B, Timmers ACJ, Emons AMC. Microtubule dynamics during preprophase band formation and the role of endoplasmic microtubules during root hair elongation. Cell Biol Int. 2003;27:295 doi: 10.1016/S1065-6995(02)00309-8 12681341 10.1016/s1065-6995(02)00309-8

[19] Vos JW, Dogterom M, Emons AM. Microtubules become more dynamic but not shorter during preprophase band formation: A possible “search-and-capture” mechanism for microtubule translocation. Cell Motil Cytoskeleton. 2004;57:246 doi: 10.1002/cm.10169 14752808 10.1002/cm.10169

[20] Dixit R, Cyr R. Encounters between Dynamic Cortical Microtubules Promote Ordering of the Cortical Array through Angle-Dependent Modifications of Microtubule Behavior. The Plant Cell. 2004;16:3274 doi: 10.1105/tpc.104.026930 15539470 10.1105/tpc.104.026930 PMC535873

[21] Wasteneys GO, Williamson RE. Reassembly of microtubules in Nitella tasmanica: quantitative analysis of assembly and orientation. Eur J Cell Biol. 1989;50:76.

[22] Kumagai F, Yoneda A, Tomida T, Sano T, Nagata T, Hasezawa S. Fate of nascent microtubules organized at the M/G1 interface, as visualized by synchronized tobacco BY-2 cells stably expressing GFP-tubulin: time-sequence observations of the reorganization of cortical microtubules in living plant cells. Plant Cell Physiol. 2001;42:723 doi: 10.1093/pcp/pce091 11479379 10.1093/pcp/pce091

[23] Ehrhardt DW, Shaw SL. Microtubule dynamics and organization in the plant cortical array. Annu Rev Plant Biol. 2006;57:859 doi: 10.1146/annurev.arplant.57.032905.105329 16669785 10.1146/annurev.arplant.57.032905.105329

[24] Lindeboom JJ, Lioutas A, Deinum EE, Tindemans SH, Ehrhardt DW, Emons AMC, et al Cortical Microtubule Arrays Are Initiated from a Nonrandom Prepattern Driven by Atypical Microtubule Initiation. Plant Physiology. 2013;161(3):1189–1201. doi: 10.1104/pp.112.204057 23300168 10.1104/pp.112.204057 PMC3585589

[25] Korn RW. The Changing Shape of Plant Cells: Transformations During Cell Proliferation. Annals of Botany. 1980;46(6):649 doi: 10.1093/oxfordjournals.aob.a085963

[26] Ambrose JC, Wasteneys GO. CLASP Modulates Microtubule-Cortex Interaction during Self-Organization of Acentrosomal Microtubules. Mol Biol Cell. 2008;19:4730 doi: 10.1091/mbc.E08-06-0665 18716054 10.1091/mbc.E08-06-0665 PMC2575154

[27] Chan J, Calder GM, Doonan JH, Lloyd CW. EB1 reveals mobile microtubule nucleation sites in Arabidopsis. Nature Cell Biol. 2003;5:967 doi: 10.1038/ncb1057 14557818 10.1038/ncb1057

[28] Dhonukshe P, Gadella TWJ. Alteration of microtubule dynamic instability during preprophase band formation revealed by yellow fluorescent protein-CLIP170 microtubule plus-end labeling. Plant Cell. 2003;15:597 doi: 10.1105/tpc.008961 12615935 10.1105/tpc.008961 PMC150016

[29] Mogilner A, Allard J, Wollman R. Cell polarity: Quantitative modeling as a tool in cell biology. Science. 2012;336:175 doi: 10.1126/science.1216380 22499937 10.1126/science.1216380

[30] Eren EC, Gautam N, Dixit R. Computer simulation and mathematical models of the noncentrosomal plant cortical microtubule cytoskeleton. Cytoskeleton. 2012;69(3):144–154. doi: 10.1002/cm.21009 22266809 10.1002/cm.21009

[31] Deinum EE, Mulder BM. Modelling the role of microtubules in plant cell morphology. Current Opinion in Plant Biology. 2013;16(6):688–692. Cell biology. doi: 10.1016/j.pbi.2013.10.001 24157061 10.1016/j.pbi.2013.10.001

[32] Tindemans SH, Hawkins RJ, Mulder BM. Survival of the Aligned: Ordering of the Plant Cortical Microtubule Array. Phy Rev Lett. 2010;104:58103 doi: 10.1103/PhysRevLett.104.058103 10.1103/PhysRevLett.104.058103 20366797

[33] Eren EC, Dixit R, Gautam N. A Three-Dimensional Computer Simulation Model Reveals the Mechanisms for Self-Organization of Plant Cortical Microtubules into Oblique Arrays. Molecular Biology of the Cell. 2010;21:2674 doi: 10.1091/mbc.E10-02-0136 20519434 10.1091/mbc.E10-02-0136 PMC2912353

[34] Ambrose C, Allard JF, Cytrynbaum EN, Wasteneys GO. A CLASP-modulated cell edge barrier mechanism drives cell-wide cortical microtubule organization in Arabidopsis. Nat Com. 2011;2:430 doi: 10.1038/ncomms1444 10.1038/ncomms1444 PMC3265373 21847104

[35] Tindemans S, Deinum E, Lindeboom J, Mulder B. Efficient event-driven simulations shed new light on microtubule organization in the plant cortical array. Frontiers in Physics. 2014;2:19 doi: 10.3389/fphy.2014.00019

[36] Gillespie DT. Exact stochastic simulation of coupled chemical reactions. The Journal of Physical Chemistry. 1977;81(25):2340–2361. doi: 10.1021/j100540a008

[37] Sedgewick R, Wayne K. Algorithms. Addison-Wesley; 2011.

[38] de Reuille PB, Routier-Kierzkowska AL, Kierzkowski D, Bassel GW, Schüpbach T, Tauriello G, et al MorphoGraphX: A platform for quantifying morphogenesis in 4D. eLife. 2015;4:e05864. 10.7554/eLife.05864 PMC4421794 25946108

[39] Cignoni P, Callieri M, Corsini M, Dellepiane M, Ganovelli F, Ranzuglia G. MeshLab: an Open-Source Mesh Processing Tool. In: Scarano V, Chiara RD, Erra U, editors. Eurographics Italian Chapter Conference. The Eurographics Association; 2008.

[40] Ueng SK, Sikorski K. A note on a linear time algorithm for constructing adjacency graphs of 3D FEA data. The Visual Computer. 1996;12:445 doi: 10.1007/BF01782476

[41] Hart JC, Francis GK, Kauffman LH. Visualizing Quaternion Rotation. ACM Transactions on Graphics. 1994;13:256 doi: 10.1145/195784.197480

[42] Osada R, Funkhouser T, Chazelle B, Dobkin D. Shape distributions. ACM Trans Graph. 2002;21:807 doi: 10.1145/571647.571648

[43] Pampaloni F, Lattanzi G, Jonáš A, Surrey T, Frey E, Florin EL. Thermal fluctuations of grafted microtubules provide evidence of a length-dependent persistence length. Proceedings of the National Academy of Sciences. 2006;103(27):10248–10253. doi: 10.1073/pnas.0603931103 10.1073/pnas.0603931103 PMC1502443 16801537

[44] Van den Heuvel MGL, de Graaff MP, Dekker C. Microtubule curvatures under perpendicular electric forces reveal a low persistence length. Proceedings of the National Academy of Sciences. 2008;105(23):7941–7946. doi: 10.1073/pnas.0704169105 10.1073/pnas.0704169105 PMC2786941 18359849

[45] Hughes JF, van Dam A, McGuire M, Sklar DF, Foley JD, Feiner SK, et al Computer graphics: principles and practice (3rd ed.). Addison-Wesley Professional; 2013.

[46] O’Rourke J. Computational Geometry in C. Cambridge University Press; 1998.

[47] Schneider PJ, Eberly D. Geometric Tools for Computer Graphics. Elsevier Science Inc.; 2002.

[48] Hawkins RJ, Tindemans SH, Mulder BM. Model for the orientational ordering of the plant microtubule cortical array. Phy Rev E. 2010;82:011911 doi: 10.1103/PhysRevE.82.011911 10.1103/PhysRevE.82.011911 20866652

[49] Deinum EE, Tindemans SH, Mulder BM. Taking directions: the role of microtubule-bound nucleation in the self-organization of the plant cortical array. Phys Biol. 2011;8:056002 doi: 10.1088/1478-3975/8/5/056002 21791726 10.1088/1478-3975/8/5/056002

[50] Deinum EE. Simple models for complex questions on plant development [PhD Dissertation (http://edepot.wur.nl/259414)]. Wageningen University; 2013.

[51] Lindeboom JJ, Nakamura M, Hibbel A, Shundyak K, Gutierrez R, Ketelaar T, et al A Mechanism for Reorientation of Cortical Microtubule Arrays Driven by Microtubule Severing. Science. 2013;342 (6163). doi: 10.1126/science.1245533 24200811 10.1126/science.1245533

[52] Atkinson S, Kirik A, Kirik V. Microtubule array reorientation in response to hormones does not involve changes in microtubule nucleation modes at the periclinal cell surface. Journal of Experimental Botany. 2014;65(20):5867 doi: 10.1093/jxb/eru325 25135522 10.1093/jxb/eru325 PMC4203123

[53] Mao T, Jin L, Li H, Liu B, Yuan M. Two Microtubule-Associated Proteins of the Arabidopsis MAP65 Family Function Differently on Microtubules. Plant Physiology. 2005;138:654–662. doi: 10.1104/pp.104.052456 15908607 10.1104/pp.104.052456 PMC1150386

[54] Fu Y, Gu Y, Zheng Z, Wasteneys G, Yang Z. Arabidopsis Interdigitating Cell Growth Requires Two Antagonistic Pathways with Opposing Action on Cell Morphogenesis. Cell. 2005;120(5):687–700. doi: 10.1016/j.cell.2004.12.026 15766531 10.1016/j.cell.2004.12.026

[55] Harries PA, Palanichelvam K, Yu W, Schoelz JE, Nelson RS. The Cauliflower Mosaic Virus Protein P6 Forms Motile Inclusions That Traffic along Actin Microfilaments and Stabilize Microtubules. Plant Physiology. 2009;149(2):1005 doi: 10.1104/pp.108.131755 19028879 10.1104/pp.108.131755 PMC2633818

[56] Sasaki T, Fukuda H, Oda Y. Cortical Microtubule Disordering is Required for Secondary Cell Wall Patterning in Xylem Vessels. The Plant Cell. 2017;. doi: 10.1105/tpc.17.00663 29133465 10.1105/tpc.17.00663 PMC5757280

[57] Sugiyama Y, Wakazaki M, Toyooka K, Fukuda H, Oda Y. A Novel Plasma Membrane-Anchored Protein Regulates Xylem Cell-Wall Deposition through Microtubule-Dependent Lateral Inhibition of Rho GTPase Domains. Current Biology. 2017;27:2522–2528.e4 doi: 10.1016/j.cub.2017.06.059 28803875 10.1016/j.cub.2017.06.059

[58] Kragler F, Curin M, Trutnyeva K, Gansch A, Waigmann E. MPB2C, a Microtubule-Associated Plant Protein Binds to and Interferes with Cell-to-Cell Transport of Tobacco Mosaic Virus Movement Protein. Plant Physiology. 2003;132:1870–1883. doi: 10.1104/pp.103.022269 12913144 10.1104/pp.103.022269 PMC181273

[59] Wang Y, Zheng X, Yu B, Han S, Guo J, Tang H, et al Disruption of microtubules in plants suppresses macroautophagy and triggers starch excess-associated chloroplast autophagy. Autophagy. 2015;11:2259–2274. doi: 10.1080/15548627.2015.1113365 26566764 10.1080/15548627.2015.1113365 PMC4835195

[60] Mineyuki Y. The Preprophase Band of Microtubules: Its Function as a Cytokinetic Apparatus in Higher Plants. International Review of Cytology. 1999;187:1–49.

[61] Müller S, Wright AJ, Smith LG. Division plane control in plants: new players in the band. Trends in Cell Biology. 2009;19(4):180–188. doi: 10.1016/j.tcb.2009.02.002 19285867 10.1016/j.tcb.2009.02.002

[62] Poethig RS, Coe EH, Johri MM. Cell lineage patterns in maize embryogenesis: A clonal analysis. Developmental Biology. 1986 10;117(2):392–404. doi: 10.1016/0012-1606(86)90308-8

[63] Marcotrigiano M, Bernatzky R. Arrangement of cell layers in the shoot apical meristems of periclinal chimeras influences cell fate. The Plant Journal. 1995 2;7(2):193–202. doi: 10.1046/j.1365-313X.1995.7020193.x

[64] Teale WD, Paponov IA, Palme K. Auxin in action: signalling, transport and the control of plant growth and development. Nature Reviews Molecular Cell Biology. 2006 11;7(11):847–859. doi: 10.1038/nrm2020 16990790 10.1038/nrm2020

[65] Vanneste S, Friml J. Auxin: A Trigger for Change in Plant Development. Cell. 2009;136(6):1005–1016. doi: 10.1016/j.cell.2009.03.001 19303845 10.1016/j.cell.2009.03.001

[66] Chan J, Sambade A, Calder G, Lloyd C. Arabidopsis cortical microtubules are initiated along, as well as branching from, existing microtubules. Plant Cell. 2009;21:2298 doi: 10.1105/tpc.109.069716 19706794 10.1105/tpc.109.069716 PMC2751946

[67] Murata T, Sonobe S, Baskin TI, Hyodo S, Hasezawa S, Nagata T, et al Microtubule-dependent microtubule nucleation based on recruitment of big gamma-tubulin in higher plants. Nat Cell Biol. 2005;7:961 doi: 10.1038/ncb1306 16138083 10.1038/ncb1306

[68] Nakamura M. Microtubule nucleating and severing enzymes for modifying microtubule array organization and cell morphogenesis in response to environmental cues. New Phytologist. 2015;205(3):1022–1027. 2014-17382. doi: 10.1111/nph.12932 25729799 10.1111/nph.12932

[69] Deinum EE, Tindemans SH, Mulder BM. Taking directions: the role of microtubule-bound nucleation in the self-organization of the plant cortical array. Physical Biology. 2011;8(5):056002 doi: 10.1088/1478-3975/8/5/056002 21791726 10.1088/1478-3975/8/5/056002

[70] Deinum EE, Tindemans SH, Lindeboom JJ, Mulder BM. How selective severing by katanin promotes order in the plant cortical microtubule array. Proceedings of the National Academy of Sciences. 2017;114(27):6942–6947. doi: 10.1073/pnas.1702650114 10.1073/pnas.1702650114 PMC5502621 28630321

[71] Uyttewaal M, Burian A, Alim K, Landrein B, Borowska-Wykret D, Dedieu A, et al Mechanical Stress Acts via Katanin to Amplify Differences in Growth Rate between Adjacent Cells in Arabidopsis. Cell. 2012;149:439 doi: 10.1016/j.cell.2012.02.048 22500806 10.1016/j.cell.2012.02.048

[72] Louveaux M, Julien JD, Mirabet V, Boudaoud A, Hamant O. Cell division plane orientation based on tensile stress in Arabidopsis thaliana. Proc Natl Acad Sci USA. 2016;113:E4294 doi: 10.1073/pnas.1600677113 27436908 10.1073/pnas.1600677113 PMC4968720

[73] Reddy JN. An Introduction to the Finite Element Method (Third ed.). McGraw-Hill; 2006.

[74] Fayant P, Girlanda O, Chebli Y, Aubin CÉ, Villemure I, Geitmann A. Finite Element Model of Polar Growth in Pollen Tubes. The Plant Cell Online. 2010;22(8):2579–2593. doi: 10.1105/tpc.110.075754 10.1105/tpc.110.075754 PMC2947179 20699395

[75] Bassel GW, Stamm P, Mosca G, Barbier de Reuille P, Gibbs DJ, Winter R, et al Mechanical constraints imposed by 3D cellular geometry and arrangement modulate growth patterns in the Arabidopsis embryo. Proceedings of the National Academy of Sciences. 2014;111(23):8685–8690. doi: 10.1073/pnas.1404616111 10.1073/pnas.1404616111 PMC4060677 24912195

[76] Long Y, Stahl Y, Weidtkamp-Peters S, Postma M, Zhou W, Goedhart J, et al In vivo FRET-FLIM reveals cell-type-specific protein interactions in Arabidopsis roots. Nature. 2017;548:97–102. doi: 10.1038/nature23317 28746306 10.1038/nature23317

[77] Musielak TJ, Schenkel L, Kolb M, Henschen A, Bayer M. A simple and versatile cell wall staining protocol to study plant reproduction. Plant Reproduction. 2015;28:161–169. doi: 10.1007/s00497-015-0267-1 26454832 10.1007/s00497-015-0267-1 PMC4623088

[78] Liu L, Zhang Y, Tang S, Zhao Q, Zhang Z, Zhang H, et al An efficient system to detect protein ubiquitination by agroinfiltration in Nicotiana benthamiana. The Plant Journal. 2010;61:893–903. doi: 10.1111/j.1365-313X.2009.04109.x 20015064 10.1111/j.1365-313X.2009.04109.x

