# Supplementary figures and images for: A computational framework for cortical microtubule dynamics in realistically shaped plant cells
Source: PLoS Comput Biol. 2018 Feb 2;14(2):e1005959. doi: 10.1371/journal.pcbi.1005959 (PMC5812663; doi:10.1371/journal.pcbi.1005959)

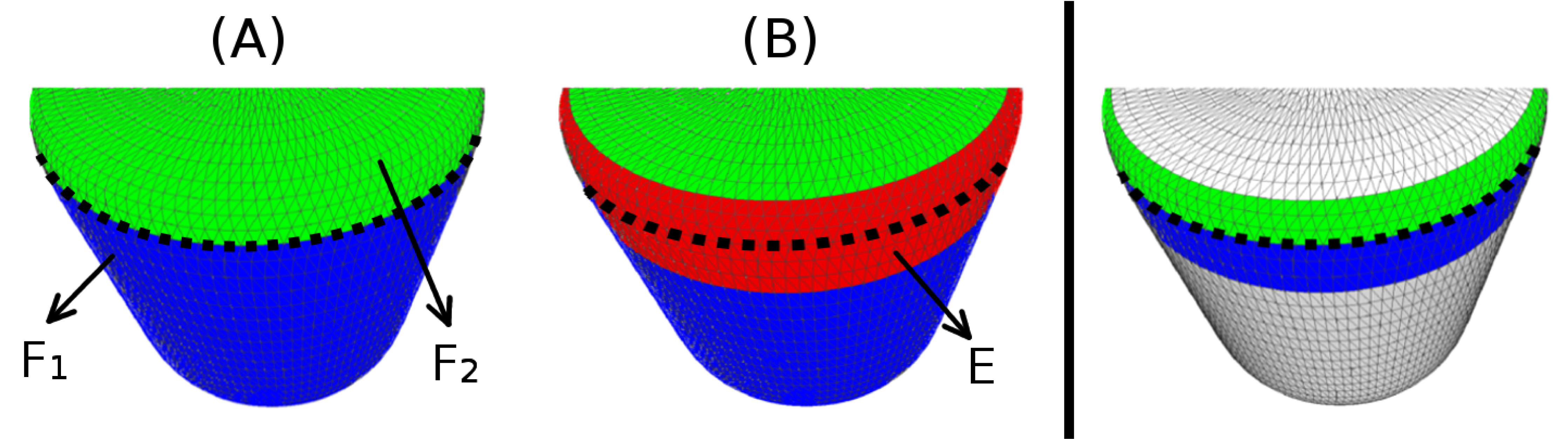

Supplement: S1 Fig — Left panel: Faces F1 (blue colour)and F2 (green colour), meet along the dotted line, (B) Using this dotted line as reference, an edge E (red colour) is detected, which is composed of multiple triangles belonging to either F1 or F2. Right panel: Triangles with edge color (ec; red) and one of the face colour (f1;blue or f2;green), are identified via the respective face colour only. (TIF) [file pcbi.1005959.s001.tif]

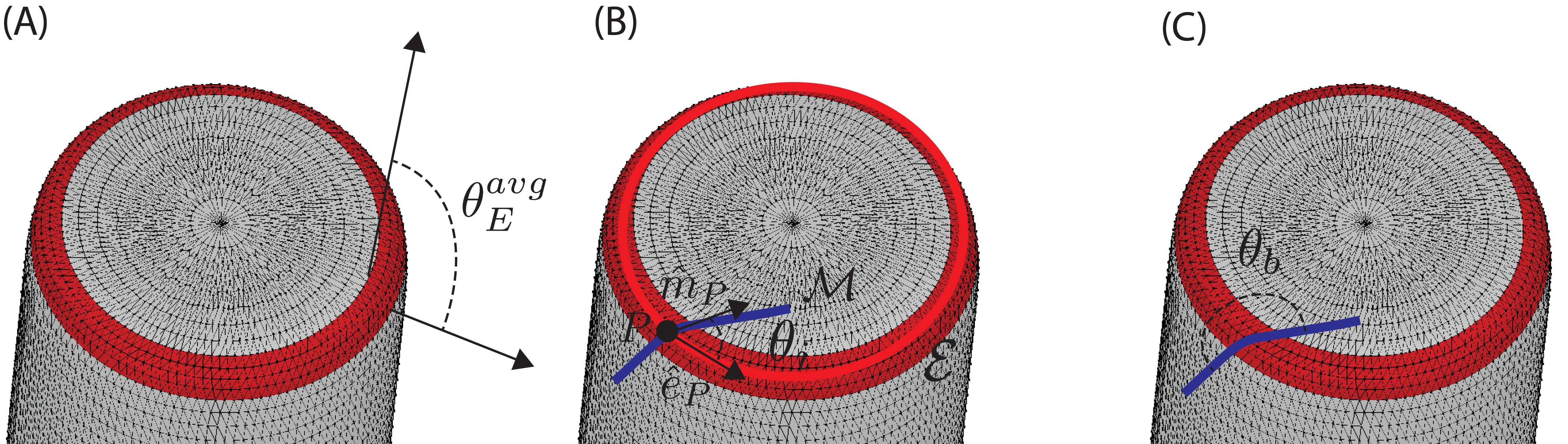

Supplement: S2 Fig — Schematic diagram of MT bending angle calculations at an edge. Left panel: (A) Edge angle θEavg, (B) Incidence angle θi: the angle between the direction of MT growth m^P along the trajectory M and the tangent e^P to the curve E of maximal curvature between the adjacent faces at the crossing point P. (C) Bending angle θb. (TIF) [file pcbi.1005959.s002.tif]

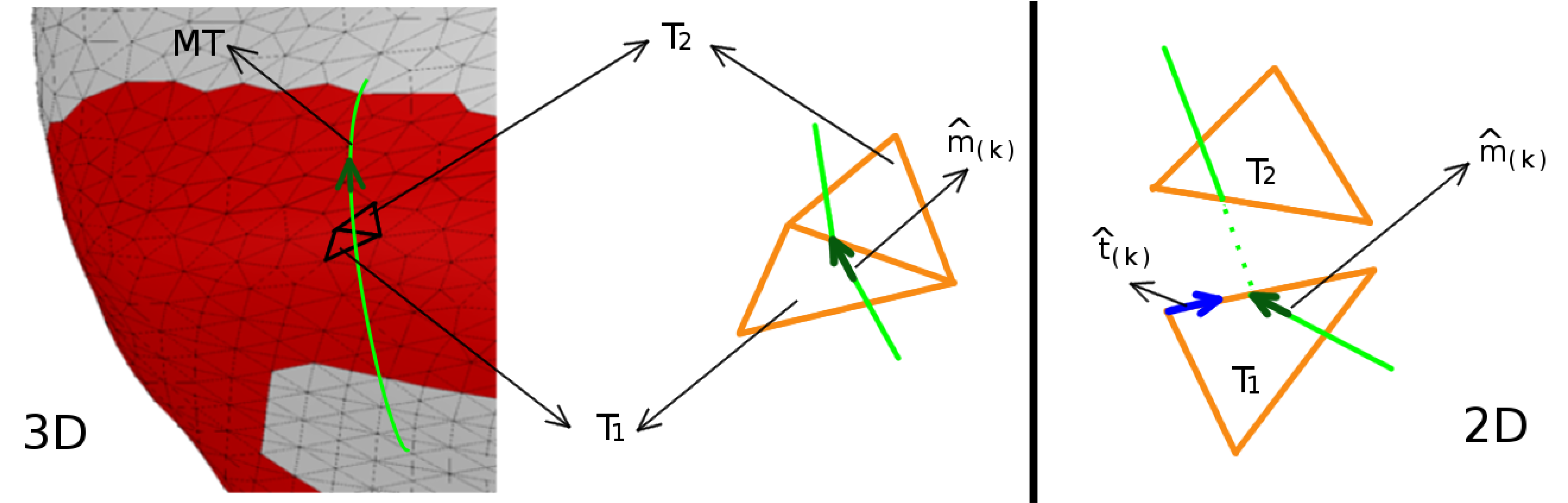

Supplement: S3 Fig — The propensity for edge-catastrophe in MT dynamics is determined through local bending of MTs through a set of triangle pairs (T1, T2), which belong to an edge (k). m^(k) is the growth direction of a MT passing from T1(k) to T2(k) through their shared edge k and t^(k) is a unit vector along this edge. (TIF) [file pcbi.1005959.s003.tif]

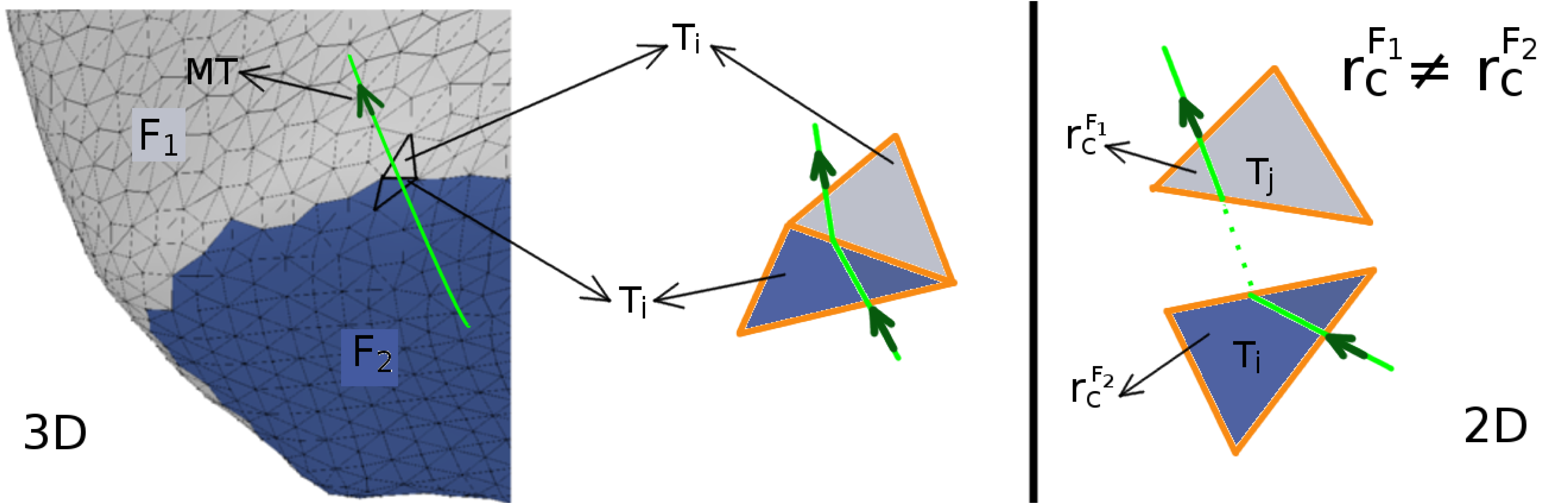

Supplement: S4 Fig — When a MT passes from a triangle Ti of a face F1 to a triangle Tj of another face F2, we update its spontaneous catastrophe from rcF1→rcF2. (TIF) [file pcbi.1005959.s004.tif]

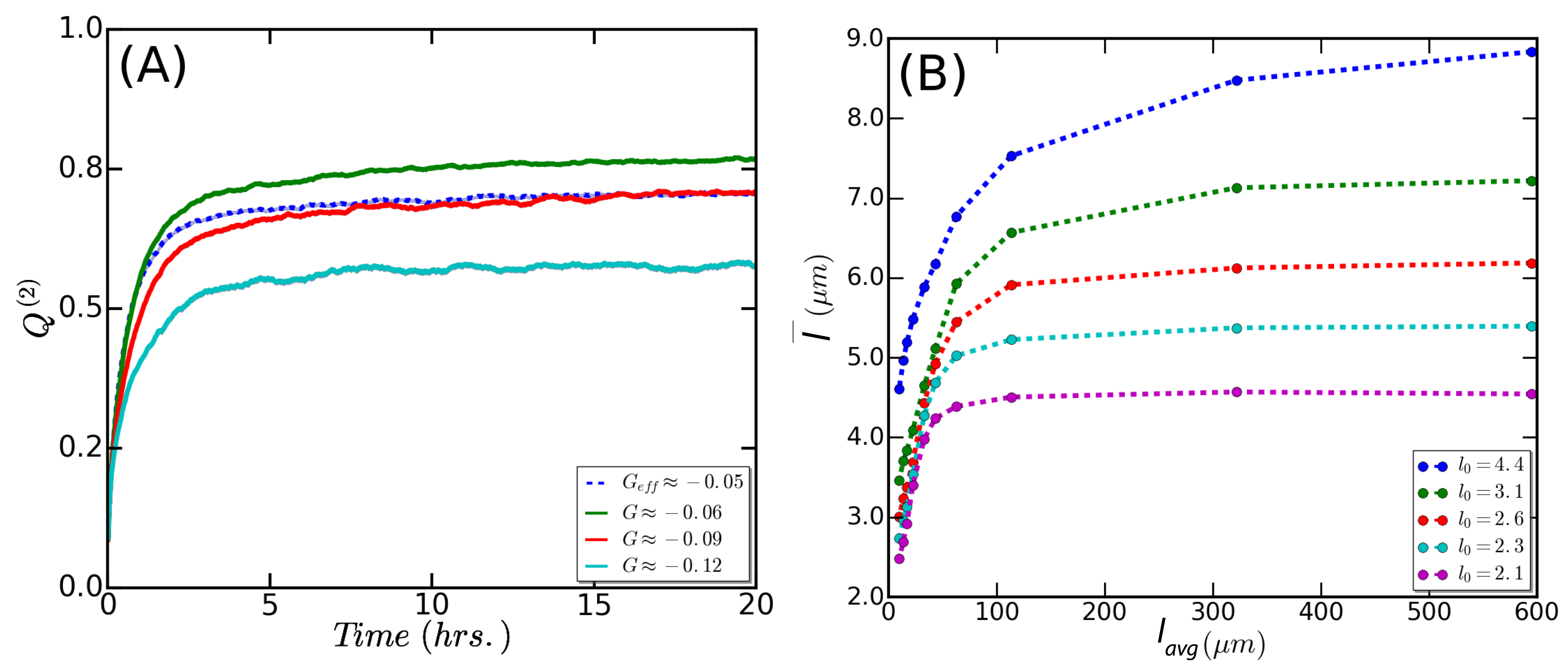

Supplement: S5 Fig — Comparison of MT ordering under an infinite tubulin pool and a finite tubulin pool and comparison between simulated (l¯) and theoretical (lavg) average MT length. (A) Time evolution of Q(2) for: (1) G with infinite tubulin pool (ρtub = ∞ μm−1), and (2) Geff with finite tubulin pool (ρtub = 10 μm−1). Due to presence of finite tubulin pool effect, calculation of G by using modified value of MT plus-end growth speed resulted in a modified value from G ≈ −0.005 to Geff ≈ −0.05. (B) For different values of l0, variation of simulated MT average length l¯ which includes interaction effects, with respect to lavg which excludes any interaction effects. (TIF) [file pcbi.1005959.s005.tif]

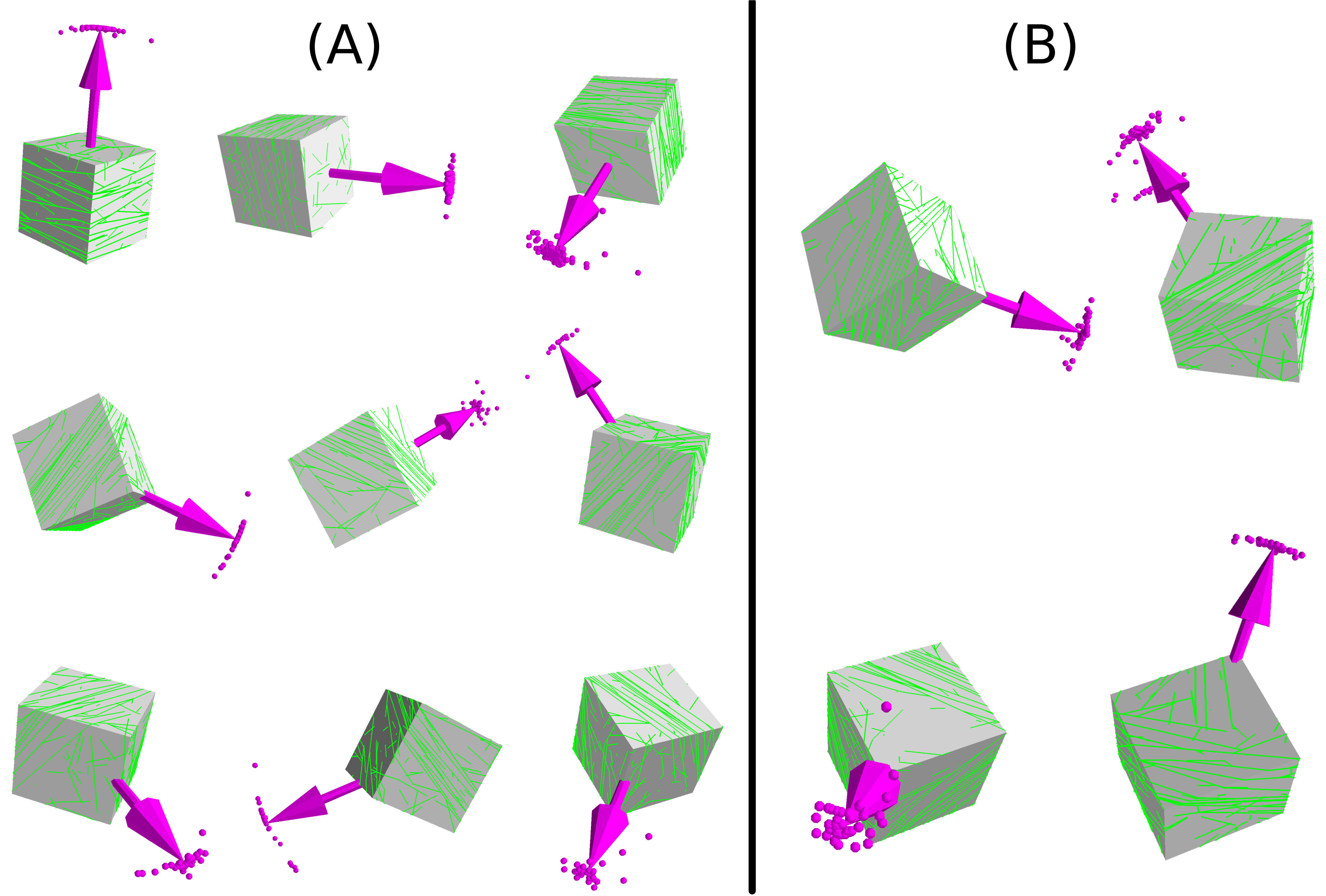

Supplement: S6 Fig — (A) Distribution of Ω^ on the surface of a sphere, triangulated by different numbers of triangles (T = 10, 30, 50, 100, 1000, 3000, 5000). (B) Distribution of Ω^ on the surface of a sphere, triangulated by different algorithms (T = I, II, III, IV) while keeping number of triangles fixed at T = 5000. (C) The Chi-squared test for homogeneity in the distribution of Ω^ tips for each case of triangulation. With the increasing number of triangles, the corresponding distribution of Ω^ tips becomes more homogeneous. (TIF) [file pcbi.1005959.s006.tif]

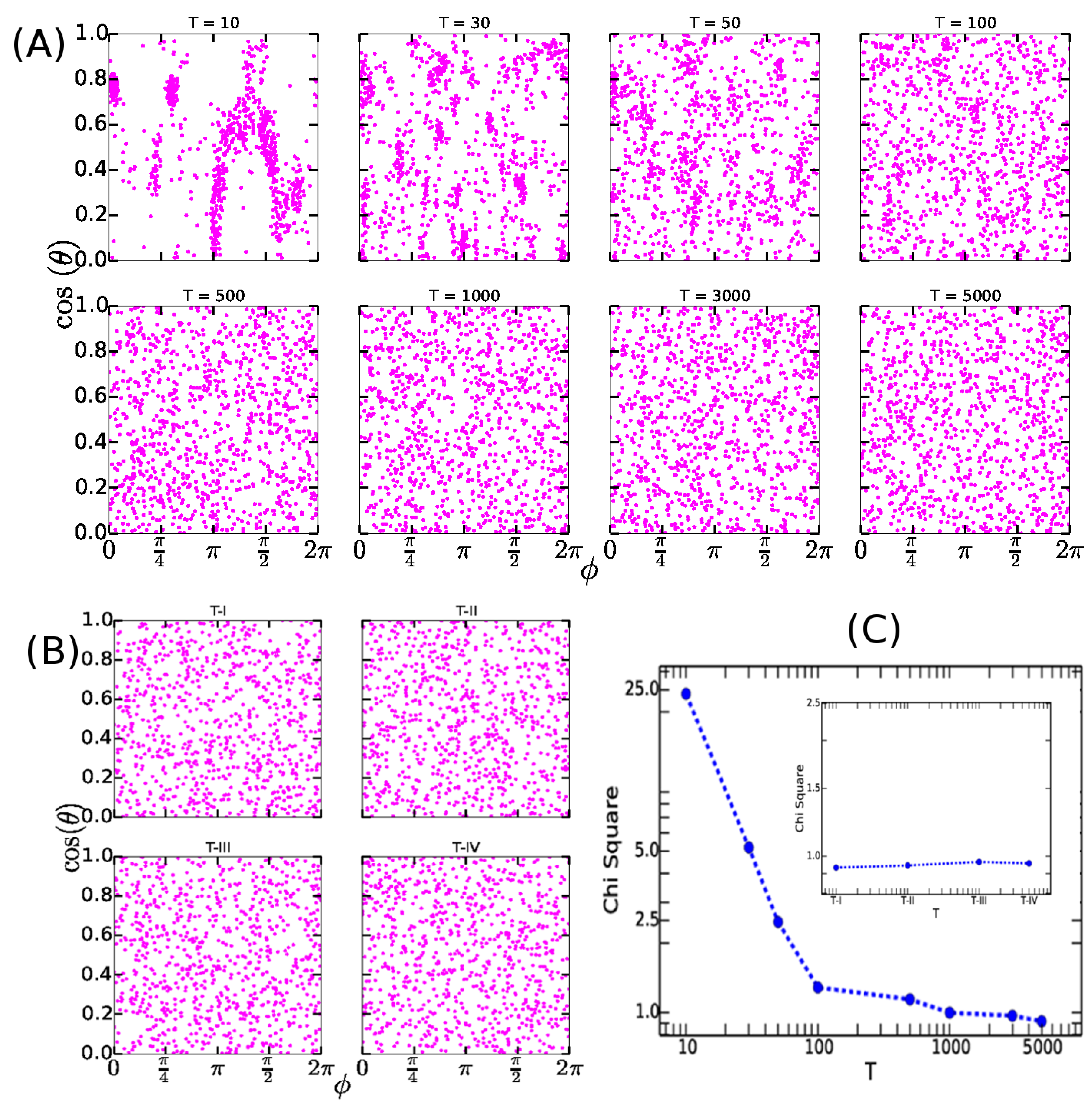

Supplement: S7 Fig — Simulated orientation of MT arrays on default cube surface with side length L = 15μm: (A) Formation of MT arrays along the nine most favoured closed geodetic paths. (B) Additionally, we also found four less-favoured paths of MT array formation, which are composed completely of diagonal paths from different faces. (TIF) [file pcbi.1005959.s007.tif]

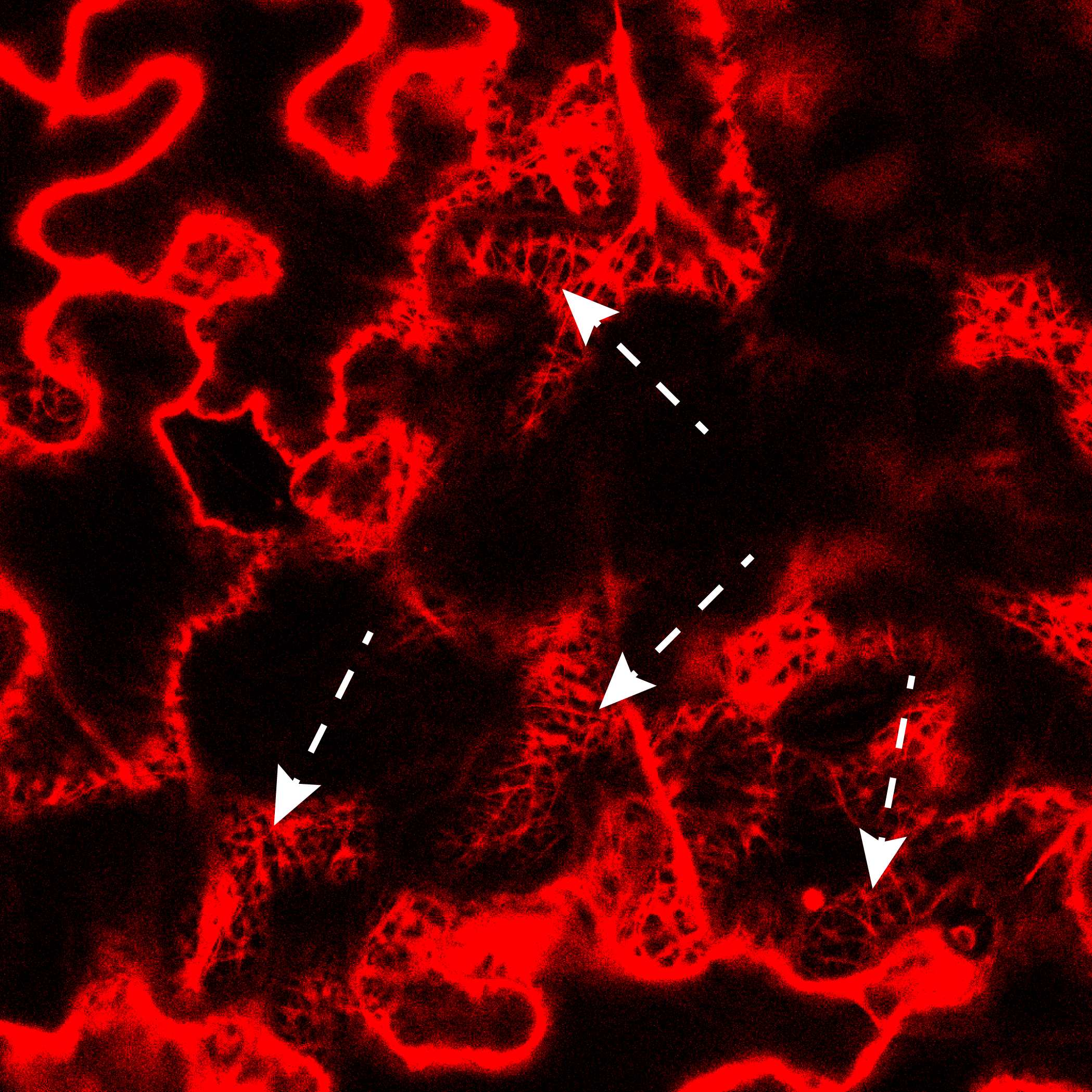

Supplement: S8 Fig — MT array pattern on the inner membrane cortex of Nicotiana benthamiana leaf pavement cell. 35S::TUB-mCHERRY lines were used to visualize the cortical MTs and ordered arrays of MTs are highlighted by the dashed arrows. (TIF) [file pcbi.1005959.s008.tif]
