# Supplementary material for: A computational framework for cortical microtubule dynamics in realistically shaped plant cells
Source: PLoS Comput Biol. 2018 Feb 2;14(2):e1005959. doi: 10.1371/journal.pcbi.1005959 (PMC5812663; doi:10.1371/journal.pcbi.1005959)
Supplement: S1 Table — Overview of the MT dynamics parameters and variables with their default values (if applicable). For description and sources see S1 File, Sec. SI.5. (PDF) [file pcbi.1005959.s009.pdf]

Simulation parameters

Parameter	Description	Value
v^+	growth speed	$0.08\mu m\ sec^{-1}$
v^-	shrinkage speed	$0.16\mu m\ sec^{-1}$
v^{tm}	treadmilling speed	$0.01\mu m\ sec^{-1}$
r_r	rescue rate	0.007 sec^{-1}
r_c	*spontaneous-catastrophe* rate	variable: 0.003-0.02 sec^{-1}
r_n	nucleation rate	variable: 0.001-0.01 $\mu m^{-2}\ sec^{-1}$
ρ_{tub}	finite tubulin pool density (length equiv.)	10 μm^{-1}
θ_z	angle of *zippering*	40^0
$p_{ind-cat}$	probability of *induced-catastrophe*	0.5
p_{cross}	probability of *crossover*	0.5
