# Supplementary material for: A computational framework for cortical microtubule dynamics in realistically shaped plant cells
Source: PLoS Comput Biol. 2018 Feb 2;14(2):e1005959. doi: 10.1371/journal.pcbi.1005959 (PMC5812663; doi:10.1371/journal.pcbi.1005959)
Supplement: S1 File — We provide the details of the definition of edge angle, the implementation of edge-catastrophes and MT stabilization, the definition of the order parameter tensor, the implementation of finite tubulin pool effects, the parametrization of the simulations, and the analysis of the effects of triangulation of the surface. (PDF) [file pcbi.1005959.s010.pdf]

Supporting Information

A Computational Framework for Cortical Microtubule Dynamics in Realistically Shaped Plant Cells

Bandan Chakrabortty, Ikran Blilou, Ben Scheres, Bela M. Mulder

SI.1 Definition of *edge angle*

Consider an edge E connecting two faces F_1 and F_2, with outward unit normals $\hat{n}_1^{(F_1)}$ and $\hat{n}^{(F_2)}$ respectively. We adopt the angle

$$\theta_E = \cos^{-1}\left(\hat{n}^{(F_1)} \cdot \hat{n}^{(F_2)}\right) \tag{SI.1}$$

as our definition of *edge angle*.

In a triangulated surface, such faces (i.e. F_1, F_2) as well as their adjacent edge (i.e. E), will be approximated by a large set of triangles. Therefore, E will be region composed of many triangles. As illustrated in Fig SI.1, we give a unique color code f_1 (blue) to

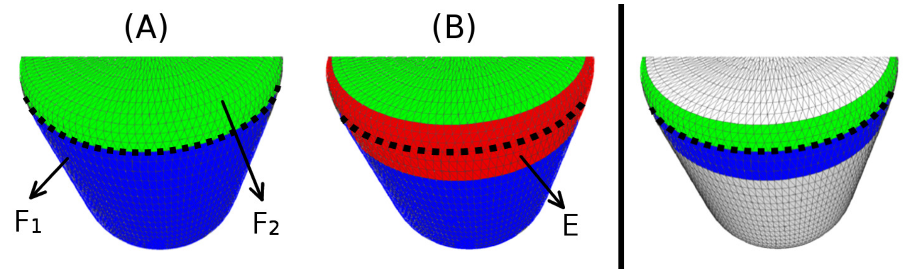

Fig SI.1. Left panel: Faces F_1 (blue colour)and F_2 (green colour), meet along the dotted line, (B) Using this dotted line as reference, an edge E (red colour) is detected, which is composed of multiple triangles belonging to either F_1 or F_2. Right panel: Triangles with edge color (e_c; red) and one of the face colour (f_1;blue or f_2;green), are identified via the respective face colour only.

the triangles that belong to F_1. Similarly, we give a unique color code f_2 (green) to the triangles that belong F_2. The triangles belonging to the edge (E) are assigned an additional color code e_c (red). Now, by our definition all the triangles with color code e_c will also have either a color code f_1 or f_2. Based on these triangles with dual color code, we calculate the average (approximate) value of the edge angle as

$$\theta_E^{avg} = \frac{\sum_{i=1}^{N_1}\sum_{j=1}^{N_2}\cos^{-1}\left(\hat{n}_i^{(f_1,e_c)} \cdot \hat{n}_j^{(f_2,e_c)}\right)}{N_1 N_2} \tag{SI.2}$$

where, $\hat{n}_i^{(f_1,e_c)}$ is the normal to the ith triangle with dual color code (f_1, e_c) and $\hat{n}_j^{(f_2,e_c)}$ is the normal to the jth triangle with dual color code (f_2, e_c). N_1 and N_2 are total number of triangles with dual color code (f_1, e_c) and (f_2, e_c), respectively.

We tested the accuracy of this approximation in calculating the edge angles for regular shapes such as cylinder, cube or isosceles pyramid etc., where the edge angles are known in advance. The calculated value of the average edge angles, θ_E^{avg} deviates only up to $\approx 5\%$ from the expected values for these shapes. Therefore, this approximation approach can be used as working formula for edge angle calculations in triangulated meshes. As the calculation of θ_E^{avg} is a a fairly laborious process, we aim to fully automate it in the future.

SI.2 Implementation of *edge-catastrophe*

Consider a MT entering an edge region E with average *edge angle* θ_E^{avg} and attempting to pass from face F_1 to face F_2. We can virtually construct its potential future trajectory $\mathcal{M}$, using the triangle-to-triangle propagation rules. To mark the actual transition point from the one face F_1 to the other face F_2, we consider a curve $\mathcal{E}$ which connects the location of maximal curvature within the edge region between F_1 and F_2, which are provided the meshing software [39]. At the intersection point P between $\mathcal{M}$ and $\mathcal{E}$ we construct the unit tangent vectors $\hat{m}_P$ and $\hat{e}_P$ to these curves (see Fig SI.2). This allows us to define the *incidence angle*

$$\theta_i = \cos^{-1}(\hat{m}_P.\hat{t}_P). \tag{SI.3}$$

The *incidence angle* determines whether the degree of curvature the MT will experience passing from the one face to the other; maximal for $\theta_i \approx \frac{\pi}{2}$ or minimal $\theta_i \approx \frac{\pi}{2}$. We then define the effective *bending angle* (θ_b) the MT experiences in passing from F_1 to face F_2 as

$$\theta_b = \sin^2(\theta_i)\theta_E^{avg}, \tag{SI.4}$$

i.e. the geometrical expression one obtains if the the smooth parts of faces F_1 and F_2 were joined directly at a sharp, cusp-like edge. This form guarantees that the maximum achievable value of the bending angle is $\theta_b^{max} = \theta_E^{avg}$, which occurs at $\theta_i = \frac{\pi}{2}$, and the minimum value is $\theta_b^{min} = 0$, which occurs at $\theta_i = 0$. This bending angle θ_b is then used as input for the actual *edge-catastrophe* calculation.

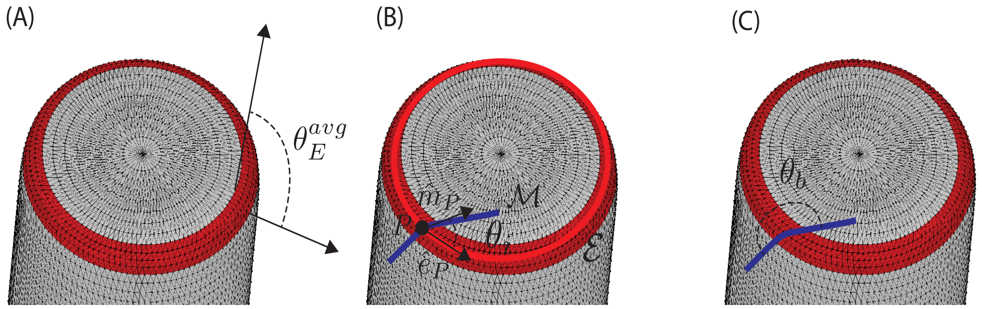

Fig SI.2. Schematic diagram of MT bending angle calculations at an edge. Left panel: (A) Edge angle θ_E^{avg}, (B) *Incidence angle* θ_i: the angle between the direction of MT growth $\hat{m}_P$ along the trajectory $\mathcal{M}$ and the tangent $\hat{e}_P$ to the curve $\mathcal{E}$ of maximal curvature between the adjacent faces at the crossing point P. (C) Bending angle θ_b.

Total *edge-catastrophe*

Geometrically speaking, for a completely flat edge $\theta_b = 0$, here MT will make a transition through the edge without any *edge-catastrophe*, i.e. probability of *edge-catastrophe* $P_E = 0$. An edge with maximum bending ($\theta_b = \pi$) will completely prohibit passage of a MT ($P_E = 1$). Based on these two extreme case scenarios, we formulate the following relation of geometry based probability of *edge-catastrophe*

$$P_E^{Geom} = \frac{1}{2}\left(1 - \cos(\theta_b)\right). \tag{SI.5}$$

However, experimental observation[26] suggests that at an edge of steep curvature with a value of $\theta_b \approx \frac{\pi}{2}$, MTs are already prohibited from crossing that edge, i.e receive full

edge-catastrophe, i.e we are allowed to set $P_E = 1$ at $\theta_b = \frac{\pi}{2}$. Accordingly we define a working form of the probability of *edge-catastrophe* (P_E^{Work})

$$P_E^{Work} = \frac{E_{cat}}{2}\left(1 - \cos(\theta_b)\right) \tag{SI.6}$$

where $0 \leqslant E_{cat} \leqslant 2$, is an *edge-catastrophe* multiplier, specific to a certain edge and its value depends on the biomechanical properties of that edge. The experimental observation that drives the foundation of Eq SI.6 is well fitted by $E_{cat} = 2$.

Local distribution of *edge-catastrophe*

For a pair of triangles $(T_1^{(k)}, T_2^{(k)})$ at their shared edge (k), we calculate the local edge angle

$$\theta_{edg}^{(k)} = \cos^{-1}(\hat{n}_1^{(k)} \cdot \hat{n}_2^{(k)}) \tag{SI.7}$$

where $\hat{n}_1^{(k)}$ and $\hat{n}_2^{(k)}$ are respective normals to $T_1^{(k)}$ and $T_2^{(k)}$, and both of the normals are pointing either inwards or outwards to the triangulated surface (see Fig SI.3).

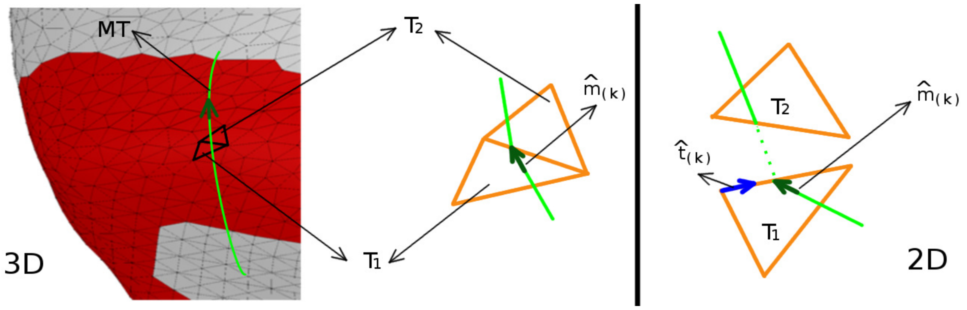

Fig SI.3. Implementing *edge-catastrophe* in MT dynamics via local bending of MTs through a set of triangle pairs (T_1, T_2), which belong to an edge (k). $\hat{m}_{(k)}$ is the growth direction of a MT passing from $T_1^{(k)}$ to $T_2^{(k)}$ through their shared edge k and $\hat{t}_{(k)}$ is a unit vector along this edge.

Considering the local *incidence angle* $\theta_i^{(k)} = \cos^{-1}(\hat{m}_{(k)}.\hat{t}_{(k)})$ (see Eq SI.3) at the edge k connecting $T_1^{(k)}$ and $T_2^{(k)}$, we define the effective local bending angle

$$\begin{aligned}\theta_b^{(k)} &= \theta_{edg}^{(k)} \sin^2(\theta_i^{(k)}) \\ &= \cos^{-1}(\hat{n}_1^{(k)} \cdot \hat{n}_2^{(k)})\left(1 - \cos^2(\theta_i^{(k)})\right) \\ &= \cos^{-1}(\hat{n}_1^{(k)} \cdot \hat{n}_2^{(k)})\left(1 - \left(\hat{m}_{(k)}.\hat{t}_{(k)}\right)^2\right)\end{aligned} \tag{SI.8}$$

where, $\hat{m}_{(k)}$ is a unit vector along the growth direction of a MT passing from $T_1^{(k)}$ to $T_2^{(k)}$ through their shared edge k and $\hat{t}_{(k)}$ is a unit vector along this edge. $\hat{n}_1^{(k)}$ and $\hat{n}_2^{(k)}$ are normals to $T_1^{(k)}$ and $T_2^{(k)}$ respectively, and both pointing either inwards or outwards to the triangulated surface.

Using Eq SI.2 and Eq SI.8, we distribute the purely geometry based total *edge-catastrophe* P_E^{Geom} (see Eq SI.5) among each pair of triangles $(T_1^{(k)}, T_2^{(k)})$ by a weight factor at their

shared edge (k)

$$
\begin{aligned}
w_{edg}^{(k)} &= \frac{\theta_b^{(k)} P_E^{Geom}}{\theta_E^{avg}} \\
&= \frac{P_E^{Geom}}{\theta_E^{avg}} \cos^{-1}(\hat{n}_1^{(k)} \cdot \hat{n}_2^{(k)}) \left(1 - \left(\hat{m}_{(k)} . \hat{t}_{(k)}\right)^2\right). \qquad \text{(SI.9)}
\end{aligned}
$$

Taking into account of the *edge-catastrophe* multiplier E_{cat} (see Eq SI.6), we define the effective local probability of *edge-catastrophe*

$$
\begin{aligned}
p_{edg}^{(k)} &= E_{cat} w_{edg}^{(k)} \\
&= \frac{E_{cat} P_E^{Geom}}{\theta_E^{avg}} \cos^{-1}(\hat{n}_1^{(k)} \cdot \hat{n}_2^{(k)}) \left(1 - \left(\hat{m}_{(k)} . \hat{t}_{(k)}\right)^2\right). \qquad \text{(SI.10)}
\end{aligned}
$$

SI.3 Implementation of MT stabilization

We illustrate the implementation technique of MT stabilization in Fig SI.4. All the triangles belonging to a face F_1 are assigned with a rate of *spontaneous catastrophe* $r_c^{F_1}$ and the triangles belonging to an adjacent face F_2 are assigned with a rate of *spontaneous*

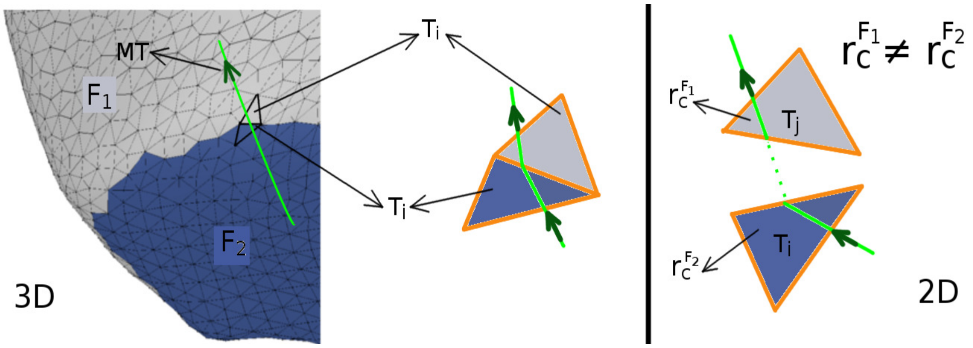

Fig SI.4. Implementing differential MT stabilization. When a MT passes from a triangle T_i of a face F_1 to a triangle T_j of another face F_2, we update its *spontaneous catastrophe* from $r_c^{F_1} \rightarrow r_c^{F_2}$.

catastrophe $r_c^{F_2}$, where $r_c^{F_1} \neq r_c^{F_2}$. In a triangle, a lower value of *spontaneous catastrophe* (r_c) results in a higher average length of the associated MT segments, leading to a longer lifetime of the segments, in comparison to another triangle with higher value of r_c. This enhancement in lifetime can be interpreted as endowing enhanced stability of the MT segments by the corresponding triangle. This way, we implement the idea of difference in stability of MTs at different faces of the triangulated surface, via face specific different rate of *spontaneous catastrophes*.

SI.4 Order parameter tensor

Consider a (piecewise) smooth oriented 2-manifold M embedded in $\mathbb{R}^3$, which we call the surface. Except for possibly a set of areal measure zero, we can endow a tangent space at each of its points σ with an orthonormal reference frame defined by two unit vectors $\hat{e}_1(\sigma)$ and $\hat{e}_2(\sigma)$, using the prescription that the outward normal to the surface is given by

$$\hat{n}(\sigma) = \hat{e}_1(\sigma) \times \hat{e}_2(\sigma).$$

At this local tangent pane, the orientation of a particle at σ can be described by a unit vector

$$\begin{aligned}\hat{\omega} &= \omega_1\ \hat{e}_1(\sigma) + \omega_2\ \hat{e}_2(\sigma) + \omega_3\ \hat{n}(\sigma) \\ &= \omega_1\ \hat{e}_1(\sigma) + \omega_2\ \hat{e}_2(\sigma) + 0\ \hat{n}(\sigma) \\ &= \cos\alpha\ \hat{e}_1(\sigma) + \sin\alpha\ \hat{e}_2(\sigma) \qquad \text{(SI.11)}\end{aligned}$$

where $\alpha\ \in [0, 2\pi)$ is the orientation angle of $\hat{\omega}$ with respect to the direction of $\hat{e}_1(\sigma)$. The local order parameter tensor $\mathbf{q}^{(m)}(\sigma)$ at σ is $O(2)$ irreducible tensor associated with rotations around $\hat{n}(\sigma)$, but now interpreted as tensors embedded in the ambient 3-space as

$$\mathbf{q}^{(m)}(\sigma) = q^{(m)}_{i_1 i_2\ \dots i_m}(\sigma) \bigotimes_{k=1,m} \hat{\mathbf{e}}_{i_k}(\sigma)$$

where $\bigotimes$ denotes direct product.

We follow the convention that lower in italic indices take on the values $\{1, 2\}$ and we adopt *Einstein summation convention.* Components of the tensor $\mathbf{q}^{(m)}(\sigma)$ upto order two are explicitly given by

$$\begin{aligned} q^{(0)}(\sigma) &= \langle 1 \rangle_\sigma\,, \\ q^{(1)}_i(\sigma) &= \langle \omega_i \rangle_\sigma\,, \\ \text{and } q^{(2)}_{ij}(\sigma) &= 2\,\langle \omega_i \omega_j \rangle_\sigma - \delta_{ij} \qquad \text{(SI.12)}\end{aligned}$$

where $\langle \dots \rangle_\sigma$ denotes orientational average, i.e. if distribution of possible orientations at σ is coded into a normalized orientational distribution $\psi_\sigma(\alpha)$, then the orientational average is

$$\langle f \rangle_\sigma = \int_0^{2\pi} d\alpha f(\alpha) \psi_\sigma(\alpha).$$

On the local tangent space, the tensor components are symmetric and traceless for $m \geq 2$.

For example

$$q^{(2)}_{ij} = \begin{bmatrix} 2\cos^2\alpha - 1 & 2\cos\alpha\sin\alpha \\ 2\cos\alpha\sin\alpha & 2\sin^2\alpha - 1 \end{bmatrix} = \begin{bmatrix} \cos 2\alpha & \sin 2\alpha \\ \sin 2\alpha & -\cos 2\alpha \end{bmatrix}.$$

we choose the normalization such that

$$q^{(m)}_{11\cdots 1}(\sigma) = \langle \cos m\alpha \rangle_\sigma.$$

We can average the local order parameter tensors over the full surface to extract the global order parameter tensor

$$\mathbf{Q}^{(m)} = \frac{\int dA(\sigma)\rho(\sigma)\mathbf{q}^{(m)}(\sigma)}{\int dA(\sigma)\rho(\sigma)} \qquad \text{(SI.13)}$$

where $\rho(\sigma)$ is the local areal density of the orientable constituents present on the elementary area dA(σ). For $m \geq 2$, symmetric and traceless properties of $\mathbf{q}^{(m)}$ remain conserved by the surface averaging process, i.e $\mathbf{Q}^{(m)}$ is also symmetric and traceless.

Extension to triangulated surface

Consider a triangulated three dimensional surface, where a bunch of rod like particles (line segments) are distributed over each triangle of the surface. For a triangle E, at its

center we assign a tangent plane with an orthonormal reference frame defined by the unit vectors $\hat{e}_1, \hat{e}_2$ and $\hat{n} = \hat{e}_1 \times \hat{e}_2$. Lets assume on the plane of this triangle, a particle of length $l_i(E)$ is oriented with angle θ_i, with respect to the direction of $\hat{e}_1$. In the local tangent frame, corresponding orientation vector (Eq SI.11) of this particle is

$$\begin{aligned}\hat{\omega}^i &= \omega_1^i \ \hat{e}_1 + \omega_2^i \ \hat{e}_2 + \omega_3^i \ \hat{n} \\ &= \cos\theta_i \ \hat{e}_1 + \sin\theta_i \ \hat{e}_2 + 0 \ \hat{n} \\ &= \cos\theta_i \ \hat{e}_1 + \sin\theta_i \ \hat{e}_2. \end{aligned} \tag{SI.14}$$

In matrix form

$$\omega^i = \begin{bmatrix}\omega_1^i \\ \omega_2^i \\ \omega_3^i\end{bmatrix} = \begin{bmatrix}\cos\theta_i \\ \sin\theta_i \\ 0\end{bmatrix}.$$

If $\mathbf{q}^{(2)}(E)$ is the local order parameter tensor associated with the triangle E, then the associated tensor components (Eq SI.12) are

$$q_{11}^{(2)}(E) = \frac{2\sum_{i=1}^{l_{max}} l_i(E)\omega_1^i\omega_1^i}{\sum_{i=1}^{l_{max}} l_i(E)} - 1,$$

$$q_{22}^{(2)}(E) = \frac{2\sum_{i=1}^{l_{max}} l_i(E)\omega_2^i\omega_2^i}{\sum_{i=1}^{l_{max}} l_i(E)} - 1,$$

$$q_{12}^{(2)}(E) = \frac{2\sum_{i=1}^{l_{max}} l_i(E)\omega_1^i\omega_2^i}{\sum_{i=1}^{l_{max}} l_i(E)} = q_{21}^{(2)}(E),$$

$$\text{and } q_{13}^{(2)}(E) = q_{31}^{(2)}(E) = q_{23}^{(2)}(E) = q_{32}^{(2)}(E) = 0$$

where l_{max} is the total number of particles present in the triangle E. We avoid explicit influence of geometry on MT order and array orientation, and also keep the resulting tensor trace less by setting $q_{33}^{(2)}(E) = 0$. In matrix form

$$\mathbf{q}^{(2)}(E) = \begin{bmatrix} q_{11}^{(2)}(E) & q_{12}^{(2)}(E) & q_{13}^{(2)}(E) \\ q_{12}^{(2)}(E) & q_{22}^{(2)}(E) & q_{23}^{(2)}(E) \\ q_{13}^{(2)}(E) & q_{23}^{(2)}(E) & q_{33}^{(2)}(E) \end{bmatrix} = \begin{bmatrix} q_{11}^{(2)}(E) & q_{12}^{(2)}(E) & 0 \\ q_{12}^{(2)}(E) & q_{22}^{(2)}(E) & 0 \\ 0 & 0 & 0 \end{bmatrix}. \tag{SI.15}$$

This local order parameter tensor $\mathbf{q}^{(2)}(E)$ is defined at the local tangent frame attached to the triangle E. To express this tensor with respect to the global coordinate frame, first we express the basis vectors $(\hat{e}_1, \hat{e}_2, \hat{n} = \hat{e}_1 \times \hat{e}_2)$ of the local tangent frame with respect to the global frame $(\hat{x}, \hat{y}, \hat{z})$ as

$$\hat{e}_1 = e_1^{(x)} \ \hat{x} + e_1^{(y)} \ \hat{y} + e_1^{(z)} \ \hat{z},$$

$$\hat{e}_2 = e_2^{(x)} \ \hat{x} + e_2^{(y)} \ \hat{y} + e_2^{(z)} \ \hat{z},$$

$$\hat{n} = n^{(x)} \ \hat{x} + n^{(y)} \ \hat{y} + n^{(z)} \ \hat{z}.$$

In matrix form

$$e_1 = \begin{bmatrix} e_1^{(x)} \\ e_1^{(y)} \\ e_1^{(z)} \end{bmatrix}, \ e_2 = \begin{bmatrix} e_2^{(x)} \\ e_2^{(y)} \\ e_2^{(z)} \end{bmatrix} \text{ and } n = \begin{bmatrix} n^{(x)} \\ n^{(y)} \\ n^{(z)} \end{bmatrix}.$$

Now, we define a rotation matrix P between the local tangent frame and the global frame

$$P = \begin{bmatrix} e_1^{(x)} & e_2^{(x)} & n^{(x)} \\ e_1^{(y)} & e_2^{(y)} & n^{(y)} \\ e_1^{(z)} & e_2^{(z)} & n^{(z)} \end{bmatrix}.$$

Using the rotational transformation, we express the local order parameter tensor $\mathbf{q}^{(2)}(E)$ with respect to the global coordinate frame as

$$\mathbf{q}_r{}^{(2)}(E) = P\mathbf{q}^{(2)}(E)P^T.$$

For all the triangles of the three dimensional polyhedral surface, averaging over the local order parameter tensors $\mathbf{q}_r{}^{(2)}$, we will get the global order parameter tensor (Eq SI.13)

$$\mathbf{Q}^{(2)} = \frac{\sum_{j=1}^{N} L_j \mathbf{q}_r{}^{(2)}(E_j)}{\sum_{j=1}^{N} L_j} \tag{SI.16}$$

where L_j is length of all the particles present on the triangle E_j and N is the total number of triangle used to approximate the polyhedral surface. In simulation, we will refer the global order parameter tensor $\mathbf{Q}^{(2)}$ as the order parameter tensor.

SI.5 Simulation parameters

Parameter	Description	Value
v^+	growth speed	$0.08\mu m\ sec^{-1}$
v^-	shrinkage speed	$0.16\mu m\ sec^{-1}$
v^{tm}	treadmilling speed	$0.01\mu m\ sec^{-1}$
r_r	rescue rate	0.007 sec^{-1}
r_c	*spontaneous-catastrophe* rate	variable: 0.003-0.02 sec^{-1}
r_n	nucleation rate	variable: 0.001-0.01 $\mu m^{-2}\ sec^{-1}$
ρ_{tub}	finite tubulin pool density (length equiv.)	10 μm^{-1}
θ_z	angle of *zippering*	40^0
$p_{ind-cat}$	probability of *induced-catastrophe*	0.5
p_{cross}	probability of *crossover*	0.5

Table 1. Overview of the MT dynamics parameters and variables with their default values (if applicable). The dynamic instability parameters were used from [19] and the value of v^{tm} has been approximated from [17]. The nucleation rate (r_n) has been chosen primarily from [33], however also varied to maintain sufficient number of MTs, such that the system always reach to an ordered state. The angle of *zippering* (θ_z) and probabilities for *zippering* (p_z), *crossovers* (p_{cross}) and *induced-catastrophes* ($p_{ind-cat}$) were deduced from [20] , combining data from MBD-DsRed and YFP-TUA6 labeling. In the biological measurements, no distinction was made between *spontaneous-catastrophe* (r_c) and *induced-catastrophe*, resulting in a likely high value by an unknown factor. Based on this observed uncertainty and the linear dependency of G on r_c, we used r_c as the primary mean to vary G.

SI.6 Finite tubulin pool effect

In most of the simulations presented in this article, we used a finite tubulin pool, which means the speed of a growing MT plus-end at any time t, is dependent on the collective total length $L(t)$ of all MTs in the system

$$v^+_{tub}(t) = v^+ \left(1 - \frac{L(t)}{L_{max}}\right) \tag{SI.17}$$

where $L_{max} = \rho_{tub}A$ is the length equivalent of the total amount of tubulin on the surface of area A and finite tubulin density ρ_{tub}, and v^+ is the simulation input MT plus-end growth speed under the assumption of an infinite tubulin pool (see Table 1).

Generically, following Eq SI.17, the speed of the growing plus-end $v^+_{tub}(t)$ will be less than v^+. Such a decrease in growth speed of MT plus-end will lead to a corresponding decrease in simulated average MT length ($\bar{l}$). As a consequence, the actual value of the control parameter (G) will tend towards a more negative value than the initially set value. First we considered an infinite tubulin pool ($\rho_{tub} = \infty\ \mu m^{-1}$) and systematically varied the value of G to achieve a steady state value of $\bar{l}$ with sufficient degree of MT order $Q^{(2)}$. As illustrated in Fig SI.5 (A), we identified a regime, $-0.06 \lessapprox G \leq -0.12$, which meet both the requirements. Here, G was calculated by using v^+, which by default assumes infinite tubulin pool. Next, we considered a system with finite tubulin pool

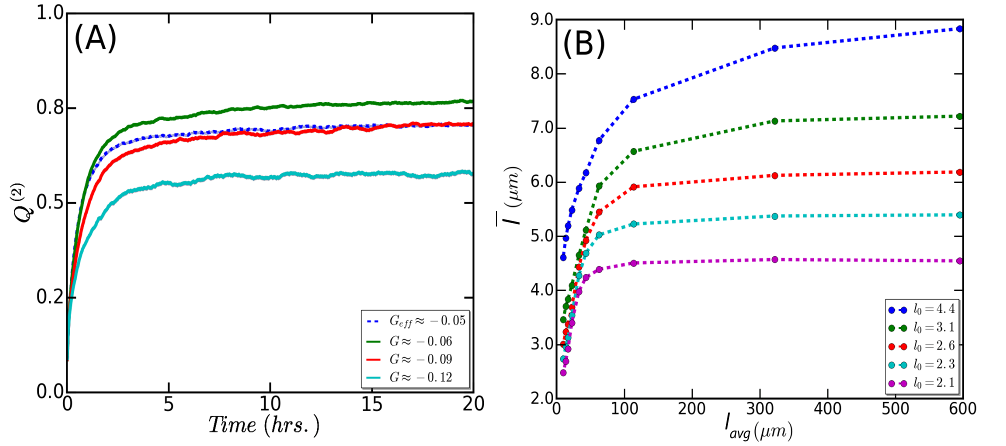

Fig SI.5. Comparison of MT ordering under an infinite tubulin pool and a finite tubulin pool and comparison between simulated ($\bar{l}$) and theoretical (l_{avg}) average MT length. (A) Time evolution of $Q^{(2)}$ for: (1) G with infinite tubulin pool ($\rho_{tub} = \infty\ \mu m^{-1}$), and (2) G_{eff} with finite tubulin pool ($\rho_{tub} = 10\ \mu m^{-1}$). Due to presence of finite tubulin pool effect, calculation of G by using modified value of MT plus-end growth speed resulted in a modified value from $G \approx -0.005$ to $G_{eff} \approx -0.05$. (B) For different values of l_0, variation of simulated MT average length $\bar{l}$ which includes interaction effects, with respect to l_{avg} which excludes any interaction effects.

($\rho_{tub} = 10\ \mu m^{-1}$) and initiated simulation of MT dynamics with $G \approx -0.005$, which was again calculated by using v^+ which assumes infinite tubulin pool. Over time the system 'eats up' the available tubulin pool, resulting in a modified value of MT plus-end growth speed from v^+ to $v^+_{tub}(t)$ (see Eq SI.17). Calculation of G by using steady state value of $v^+_{tub}(t = T_{max})$ resulted in a modified value from $G \approx -0.005$ to $G_{eff} \approx -0.05$, where both corresponds to the set of same input MT parameter values and is the working domain in Fig 10. T_{max} sets the upper limit of simulation time which assured steady state MT order and array formation. The interesting point to note here is the value of control parameter $G_{eff} \approx -0.05$ with finite tubulin pool to be equivalent to that with infinite tubulin pool, i.e. $-0.06 \lessapprox G \leq -0.12$, suggesting that effective value of working domain remains invariant under the assumption of finite tubulin pool. In Fig 10, the values of l_{avg} which are in order of $10^2 \mu m$, may at first sight seem unrealistically large. They are, however, the *bare* (theoretical) values in the absence of both pool limitations effects, as well as additional *induced-catastrophes* by the MT interactions. Here, they are used solely for the purposes of establishing a proper parametrization of the simulations in which all the relevant effects are included. The actual simulated average MT length ($\bar{l}$) are in the order of a few μm's, as illustrated in Fig SI.5 (B).

SI.7 Triangulation effect on MT-array orientation distribution

In terms of the polar angles (θ, ϕ) the infinitesimal element of surface on the sphere is given by $dS(\theta, \phi) = \sin\theta d\theta d\phi$. Introducing the cartesian coordinates

$$\begin{aligned} x &= \cos\theta \\ y &= \phi, \end{aligned} \quad \text{(SI.18)}$$

we see that the infinitesimal element of surface area in terms of these coordinates becomes the Euclidean area measure $dS(x, y) = dxdy$. We can therefore homogeneously map the surface of the sphere to the rectangular planar domain $[-1, 1] \times [0, 2\pi] \in \mathbb{R}^2$. Using

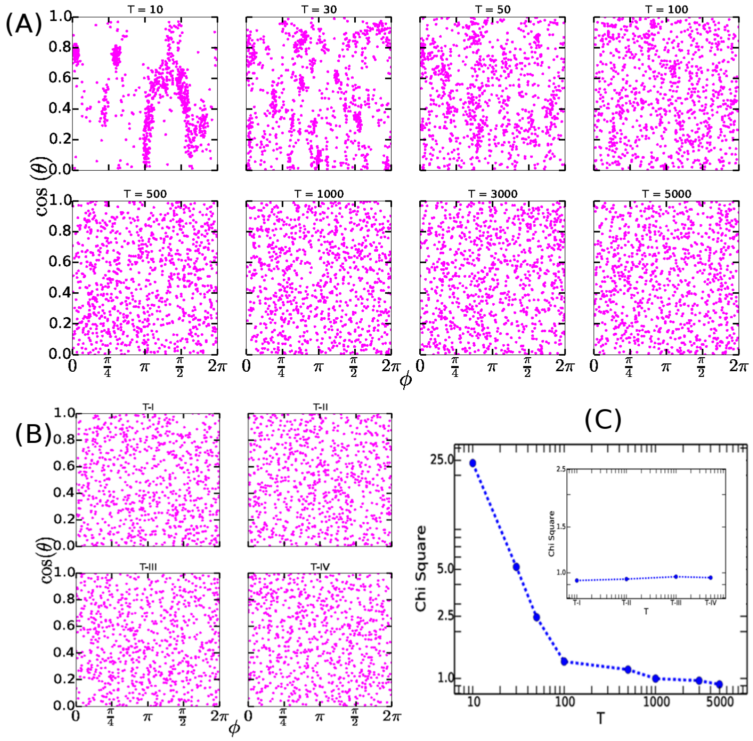

Fig SI.6. (A) Distribution of $\hat{\Omega}$ on the surface of a sphere, triangulated by different numbers of triangles ($T = 10, 30, 50, 100, 1000, 3000, 5000$). (B) Distribution of $\hat{\Omega}$ on the surface of a sphere, triangulated by different algorithms ($T = I, II, III, IV$) while keeping number of triangles fixed at $T = 5000$. (C) The Chi-squared test for homogeneity in the distribution of $\hat{\Omega}$ tips for each case of triangulation. With the increasing number of triangles, the corresponding distribution of $\hat{\Omega}$ tips becomes more homogeneous.

meshlab [39], in Fig SI.6(A), we triangulated a sphere of radius $r \approx 6\ \mu m$ by using eight different numbers of triangles ($T = 10, 30, 50, 100, 1000, 3000, 5000$). For Fig SI.6 (B), we triangulated the sphere using four different triangulations, while keeping the number of triangle fixed at $T = 5000$. The differences are obtained by inducing stochasticity in the individual triangle shapes, by randomizing the maximum desirable triangle area in the algorithm.

SI.8 MT simulation on default cube surface

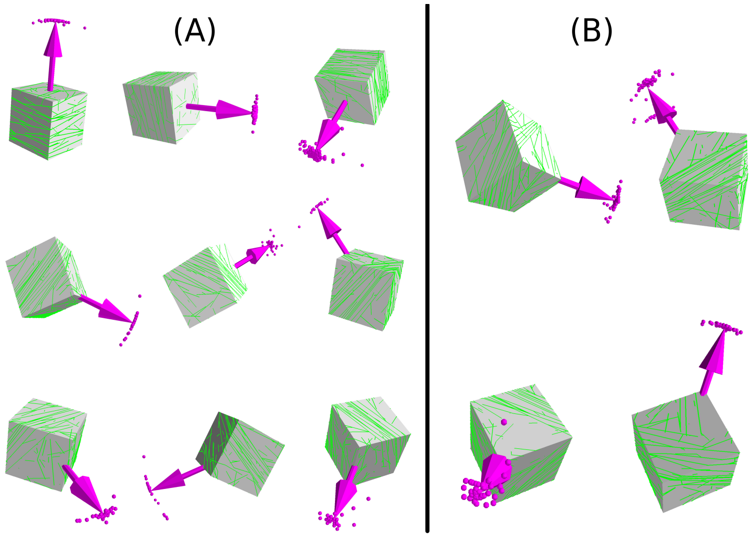

Fig SI.7. Simulated orientation of MT arrays on default cube surface with side length $L = 15\mu m$: (A) Formation of MT arrays along the nine most favoured closed geodetic paths. (B) Additionally, we also found four less-favoured paths of MT array formation, which are composed completely of diagonal paths from different faces.

SI.9 MTs on *Nicotiana benthamiana* leaf pavement cell cortex

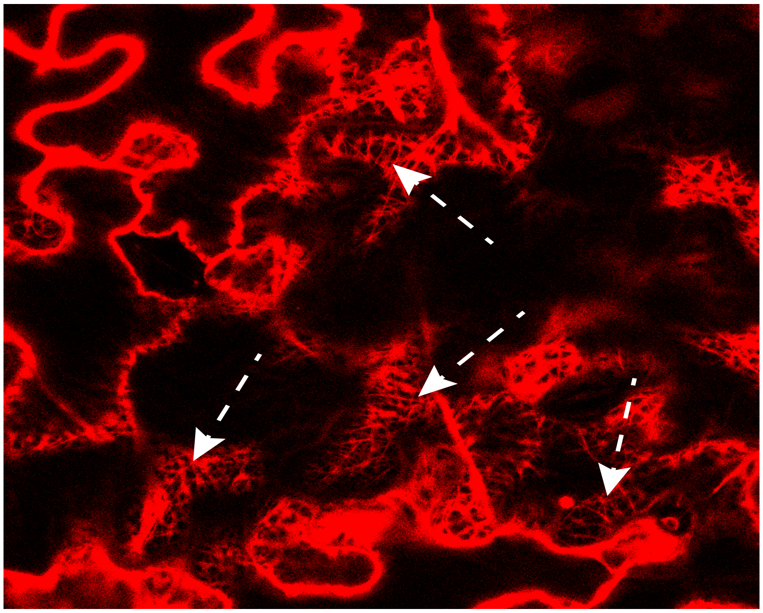

Fig SI.8. Experimental observation of MT array pattern on the inner membrane cortex of *Nicotiana benthamiana* leaf pavement cell. 35S::TUB-mcherry lines were used to visualize the cortical MTs and ordered arrays of MTs are highlighted by the dashed arrows.
